# The role of nanomaterials in enhancing natural product translational potential and modulating endoplasmic reticulum stress in the treatment of ovarian cancer

**DOI:** 10.3389/fphar.2022.987088

**Published:** 2022-10-26

**Authors:** Rajeev K. Singla, Pooja Sharma, Dinesh Kumar, Rupesh K. Gautam, Rajat Goyal, Christos Tsagkaris, Ankit Kumar Dubey, Himangini Bansal, Rohit Sharma, Bairong Shen

**Affiliations:** ^1^ Institutes for Systems Genetics, Frontiers Science Center for Disease-Related Molecular Network, West China Hospital, Sichuan University, Chengdu, China; ^2^ School of Pharmaceutical Sciences, Lovely Professional University, Phagwara, India; ^3^ Department of Pharmaceutical Sciences and Drug Research, Punjabi University, Patiala, India; ^4^ Khalsa College of Pharmacy, Amritsar, India; ^5^ Chitkara University School of Pharmacy, Chitkara University, Himachal Pradesh, India; ^6^ Department of Pharmacology, Indore Institute of Pharmacy, IIST Campus, Opposite IIM Indore, Indore, India; ^7^ MM College of Pharmacy, Maharishi Markandeshwar (Deemed to be University), Mullana-Ambala, India; ^8^ Faculty of Medicine, University of Crete, Heraklion, Greece; ^9^ iGlobal Research and Publishing Foundation, New Delhi, India; ^10^ Delhi Institute of Pharmaceutical Sciences and Research, New Delhi, India; ^11^ Department of Rasa Shastra and Bhaishajya Kalpana, Faculty of Ayurveda, Institute of Medical Sciences, BHU, Varanasi, India

**Keywords:** ovaries, natural compounds, ovarian neoplasms, nanotechnology, phytomedicine, ER stress, nanomaterial, secondary metabolites

## Abstract

Ovarian cancer, and particularly its most frequent type, epithelial ovarian carcinoma, constitutes one of the most dangerous malignant tumors among females. Substantial evidence has described the potential of phytochemicals against ovarian cancer. The effect of natural compounds on endoplasmic reticulum (ER) stress is of great relevance in this regard. In ovarian cancer, the accumulation of misfolded proteins in the ER lumen results in decompensated ER stress. This leads to deregulation in the physiological processes for the posttranslational modification of proteins, jeopardizes cellular homeostasis, and increases apoptotic signaling. Several metabolites and metabolite extracts of phytochemical origin have been studied in the context of ER stress in ovarian cancer. Resveratrol, quercetin, curcumin, fucosterol, cleistopholine, fucoidan, and epicatechin gallate, among others, have shown inhibitory potential against ER stress. The chemical structure of each compound plays an important role concerning its pharmacodynamics, pharmacokinetics, and overall effectiveness. Studying and cross-comparing the chemical features that render different phytochemicals effective in eliciting particular anti-ER stress actions can help improve drug design or develop multipotent combination regimens. Many studies have also investigated the properties of formulations such as nanoparticles, niosomes, liposomes, and intravenous hydrogel based on curcumin and quercetin along with some other phytomolecules in ovarian cancer. Overall, the potential of phytochemicals in targeting genetic mechanisms of ovarian cancer warrants further translational and clinical investigation.

## Introduction

Ovarian cancer, a deadly and malignant cancer in females, is of multiple types, but epithelial ovarian carcinoma accounts for majority of the cases (Ho et al., 2012; Khanra et al., 2012). The key factors impacting the proper prognosis of ovarian cancer are diagnosis at an advanced stage and 1° or 2° drug resistance, and all these factors overall reduced the 5-year survival rate to just 30% (Siddik, 2003). Ovarian cancer cells have the tendency to establish a resistance to common cancer therapies. Cancer cells can acquire drug resistance *via* multiple mechanisms (Ventura et al., 2007; Shafabakhsh and Asemi, 2019). Wide experimental studies have demonstrated that phytochemicals exert significant potential against ovarian cancer (Tian et al., 2017).

Since ancient times, nature has been a wonderful source of sustainable bioresources, yielding many therapeutically active compounds, including those for the treatment and management of cancer (Shen and Singla, 2020; Singla, 2021; Singla et al., 2021; Singla et al., 2022a; Singla et al., 2022b; Singla et al., 2022c; Kumar et al., 2022). This makes food, medicinal plants, and dietary supplements a goldmine and reservoir to discover promising and novel therapeutic molecules (Amin et al., 2011; Amin et al., 2021; Juaid et al., 2021). Furthermore, the synergistic potential is one of the most common observations concerning natural products, which is probably why the crude extract or enriched fractions most of the time exerts a superlative effect than the pure molecules (Rasoanaivo et al., 2011; El-Dakhly et al., 2020). Liu and the collaborators have compiled the studies of the natural products eliciting effects against endoplasmic reticulum (ER) stress, and provided information for the natural products active against ER stress-related apoptosis, reactive oxygen species (ROS)-mediated ER stress, calcium-mediated ER stress, inflammation-mediated ER stress, and ER-stress-related autophagy (Liu et al., 2016).

In this review, we will examine some classes of natural compounds showing the ability to induce ER stress-related death in cancer cells. In particular, we will focus on the ER stress-related anti-ovarian cancer activity exerted by natural compounds and also the impact of nanoformulations in the augmentation of their efficacy and bioavailability.

## Pathophysiology of ovarian cancer

Epithelial ovarian carcinoma (EOC) is a life-threatening illness, for which a specific cause is unknown. Despite enormous ideas on the genesis of ovarian cancer, additional studies are needed to explicitly explore the functions of hormonal, inflammatory, and immunological causal factors (Brett et al., 2017). Historically, the ovarian surface epithelium was believed to be the leading cause of ovarian cancer. Similarly, the idea of incessant menstruation holds that repeated engagement of the ovarian surface in the ovulation mechanism is a potential risk for ovarian cancer (Kurman and Shih, 2010). One of the most difficult aspects of understanding the etiology of ovarian cancer is that it is a heterogeneous illness consisting of several kinds of tumors having significantly disparate clinical and pathological characteristics and behavior (Lalwani et al., 2011).

Ovarian tumors differ from several other malignancies, which usually intermittently spread outside of the peritoneum. When cells from the original tumor detach and migrate into the peritoneum, they implant into the mesothelial lining, leading ovarian cancers to expand into the peritoneal cavity. Although lung metastasis is present at the time of diagnosis, further peritoneum metastasis is typically reserved for recurrent or severe disease (Cannistra et al., 1993; Lengyel, 2010; Orsulic et al., 2014). Furthermore, the recent identification of ovarian cancer stem cells in the peritoneal cavity that exhibit features of normal cancer stem cells is a novel potential contributor to both chemoresistance and metastasis (Zong and Nephew, 2019).

The etiology of certain ovarian cancers, particularly surface epithelial tumors, has been best defined because of genetic and molecular research. Serous tumors have been discovered to lack heterozygosity on chromosome 17q. Endometrioid and clear cell carcinomas can be caused by previous endometriosis, according to allelic research (Garcia et al., 2000; Baxter, 2001). The peritoneum or serosal surfaces are involved in around 80% of the more frequent epithelial ovarian malignancies as microscopic foci and visible lesions. The metastases may be exophytic, requiring direct contact with the peritoneal cavity and its contents, or sub-peritoneal foci that coalesce over time to create plaque-like deposits of varying sizes (Freedman et al., 2004; Halkia et al., 2012). Although peritoneal and serosal implantations are common in epithelial ovarian cancer, little is understood about how the multi-structured peritoneum contributes to infiltration, metastases, and tumor growth. Critical alterations in the peritoneum and stromal surfaces may accompany either capillary or hematogenous dissemination to distant locations, albeit this is unlikely (Mikuła-Pietrasik et al., 2017).

Researchers developed a dual paradigm that divides diverse kinds of ovarian cancer into two categories, type I and type II, based on a number of morphometric and molecular genetic investigations (Shih and Kurman, 2004). To accurately distinguish between type I and type II ovarian carcinoma; histopathologic, clinical, and molecular genetic profiles were effectively used. Clear cell, endometrioid, low-grade serous carcinomas, mucinous carcinomas, and malignant Brenner tumors are examples of type I ovarian cancers that grow from benign precursor lesions implanted in the ovary (Prat, 2012). Because type I carcinomas have multiple mutations in protein kinases, the mechanisms they regulate may be susceptible to inhibitor treatments or biologics. KRAS and BRAF mutations, which trigger the carcinogenic mitogen-activated protein kinase (MAPK) signaling pathway, are by far the most prevalent genetic mutations identified in type I carcinomas (Shih and Kurman, 2004). Mutations in either the KRAS or BRAF genes, which are MAPK’s upstream regulators, trigger constitutive activation of the MAPK signaling pathway in many type I carcinomas (Kurman and Shih, 2010).

Type II ovarian tumors arise from intraepithelial carcinomas of the fallopian tube and can spread to the ovary and other locations, such as high-grade serous carcinomas, which are divided into morphologic and molecular subgroups (Vang et al., 2009). Apart from these malignancies, invasive heterogeneous mesodermal tumors (carcinosarcomas) are also classified as type II since they include epithelial characteristics that are equivalent to those of pure type II carcinomas. These carcinomas are particularly aggressive and nearly arise usually at a later phase. KRAS and BRAF mutations are found in 65% of serous borderline tumors (SBTs) but are uncommon in high-grade serous carcinoma. KRAS mutations can also be found in 60% of mucinous carcinomas, 5–16% of clear cell carcinomas, and 4% of endometrioid type II carcinomas. PTEN mutations, which cause constitutive PI3K signaling, are observed in 20% of type I endometrioid neoplasms (Sieben et al., 2004; Ji et al., 2007; Karst and Drapkin, 2010). Ovarian carcinoma has, indeed, been mistakenly viewed as a single illness since it takes into account 75% of all epithelial ovarian carcinomas, has very comparable morphologic characteristics, and has a consistently dismal prognosis (McCluggage, 2002; Cho and Shih, 2009).

The EGF/ErbB family of tyrosine kinase receptors is recognized to be important in proper primordial follicle formation and in controlling the growth of the ovarian surface epithelium; the ErbB expression profiles deliver scientific proof for such functional activities. So far, reports revealed that the ErbB family of tyrosine kinase receptors and associated ligands is implicated in the genesis and development of epithelial ovarian cancer (Maihle et al., 2002; Lafky et al., 2008). ErbB receptors have a crucial physiological role in normal cellular proliferation, development, division, metabolic activity, locomotion, longevity, malignant cell expansion, adherence and cell migration, tumorigenesis, and mortality (Holbro, 2003; Appert-Collin et al., 2015). As a result, it is not unexpected that abnormalities in ErbB receptor signal transduction pathways, such as gene transcription, mutations, receptor overexpression, and the ligand-increased expression, can result in enhanced cellular proliferation and cellular transformation of epithelial cells (Wang, 2017). Indeed, ErbB gene amplification and/or ErbB receptor overexpression has been found in a wide range of human carcinomas, together with ovarian cancer (Wee and Wang, 2017).

## Cytokine storm and ER stress

Tumor growth and development are influenced by cytokine expression within the tumor microenvironment; tumor cells that generate immunosuppressive cytokines can evade the specific immune system. Numerous cytokines involved in cell-mediated immunity, including IFN-γ, IL-6, MHC, and TNF-α molecules, have been associated with increased growth and patient prognosis in ovarian cancer (Nelson, 2008; Yanaihara et al., 2012). Cancerous cells release IL-6 within the ovarian cancer microenvironment, resulting in limitation of dendritic cell maturation and promoting immunosuppressive classically activated tumor-associated macrophages (TAMs), compromising the stimulation of tumor-infiltrating T cells. IL-6-producing myeloid-derived suppressor cells (MDSCs), on the other hand, inhibit CD4^+^ T cell Th1 differentiation, reducing their capacity to assist CD8^+^ T cells and dendritic cells, resulting in poorer adaptive immunity against the development of ovarian cancer (Nishio et al., 2014; Luo et al., 2021).

The potential of IL-6 to enhance tumor growth and progression by autocrine and paracrine effects and to produce particular immunological and metabolism changes that influence prognosis has been convincingly shown in ovarian cancer (Berek et al., 1991; Lo et al., 2011). Cytokines and bioactive lipids are abundant in the ovarian tumor microenvironment. Ovarian cancer patients have been found to have a higher concentration of inflammatory cytokines such as IL-6, CCL2/MCP-1, and TNF-α, which play an important role in the disease’s genesis and progression. These cytokines feed a negative cycle by attracting and stimulating cytokine-producing cells, which then enhance their protumorigenic function even more (Gastl and Plante, 2000; Macciò et al., 2021).

According to the latest scientific studies, cytokine antagonists might be used to cure ovarian cancer. In patients with platinum-resistant ovarian cancer, however, targeting a specific cytokine/chemokine (e.g., monoclonal antibodies against IL-6 or TNF-α) has only shown a transient and limited antitumor effect. As a result, inhibiting only one or a few cytokines produced by cytotoxic therapy-induced debris would not be enough to inhibit tumorigenesis (Sandhu et al., 2013; Gartung et al., 2019). IL-6 is a key stimulatory cytokine found in the milieu of ovarian cancer, and IL-6 inhibitors may be used to treat the disease. The pleiotropic effects of IL-6 can impact almost every organ. IL-6 affects the health prognosis by changing metabolic activity, hematopoiesis, and nutritional requirements and also producing significant endothelium injury, as seen in ovarian cancer (Coward et al., 2011; Macciò and Madeddu, 2012).

Chemokines are by far the most numerous categories of cytokines, and they have been classified as CC chemokines, CXC chemokines, C chemokines, and CX3C chemokines depending on the characteristics of the first two cysteine residues (Vilgelm and Richmond, 2019). As essential mediators of the inflammatory response, they play a significant role in tumor development and metastasis. Katrina et al. found that high levels of STAT1 and STAT1 target genes (CXCL9, CXCL10, and CXCL11) are substantially associated with better treatment response in ovarian cancer (Au et al., 2016). It is extensively documented that tumorigenesis creates high levels of ROS to stimulate proximal signaling pathways that promote proliferating, longevity, and physiological adaptability, even while ensuring a high degree of antioxidant properties to avoid ROS accumulation levels that might cause cell damage (Zorov et al., 2014). Ovarian cancer is regarded as an excellent tumorigenesis-based malignancy since its cells have no detrimental influence on immune cells. Several studies and clinical trials are underway to develop effective immunotherapy for ovarian cancer. Current cancer immunotherapies encompass therapeutic vaccines, cytokine, immunological modulators, immune checkpoint inhibitors, and adoptive T cell transfer (Kandalaft et al., 2011; De Felice et al., 2015).

On analyzing single-cell and serous EOC patient samples to healthy tissues, studies reported the ER stress-related proteins ATF6, GRP78, and PERK are significantly expressed, suggesting that ER stress pathways play a role in EOC. These proteins’ expression correlated with the tumor stage, indicating that they play a role in EOC development. Also, researchers reported that increasing levels of GRP78 and PDI are attributed to lower survival rates in patients with elevated serous type EOC. Finally, we argue that the combination of GRP78 and PDI is an independent predictive factor for EOC (Qu et al., 2013; Samanta et al., 2020).

## Natural inhibitors alleviating endoplasmic reticulum stress for management of ovarian cancer: metabolite extracts and metabolites

In clinical practice, debulking surgery is the first-line treatment for resectable ovarian cancer, followed by platinum coupled with paclitaxel chemotherapy. Challenges associated with this treatment strategy include multidrug resistance, high rate of relapse, drug allergy, and paclitaxel-induced peripheral neuropathic pain (Ma et al., 2019). Thus, there is an urgent need to produce novel medications to improve the management of ovarian cancer (Wang et al., 2019).

The endoplasmic reticulum (ER) is a compartment of eukaryotic cells that is responsible for protein synthesis, folding, and trafficking into the secretory pathway (Boot-Handford and Briggs, 2009). The ER exhibits an imperative role in the execution of several cellular processes that are needed for regular cellular functions. The accretion of unfolded proteins in the endoplasmic reticulum prompted ER stress and subsidizes the advancement of several disorders such as cardiovascular diseases, neurodegenerative diseases, diabetes, obesity, cancer, atherosclerosis, stroke, liver diseases, and immune disorders (Abdullah and Ravanan, 2018).The UPR (unfolded protein response ) solicits to mitigate the protein loading in the endoplasmic reticulum *via* synchronizing a sequential block in the translation of proteins and triggers the sequence of gene transcriptional programming to increase the folding capacity of the endoplasmic reticulum (Wang G et al., 2017). UPR-associated sensors such as IRE1α (inositol-requiring enzyme-1α), ATF6 (activating transcription factor-6), GADD153 (growth arrest and DNA damage-inducible gene 153), PERK (PKR-like ER kinase), and GRP78 (glucose regulatory protein) help sustain the homeostasis of the cellular system (Liu et al., 2016). Peculiar triggers for ER stress comprise certain intracellular amendments (such as calcium or redox imbalances), some prescription medications (such as celecoxib and nelfinavir), microenvironmental circumstances (such as acidosis, hypoglycemia, and hypoxia), a high-fat diet, and several natural products (Schönthal, 2012). Recent studies have implied that ER stress endorses cell apoptosis and is associated with follicular atresia (Wu et al., 2018).

**FIGURE 1:**
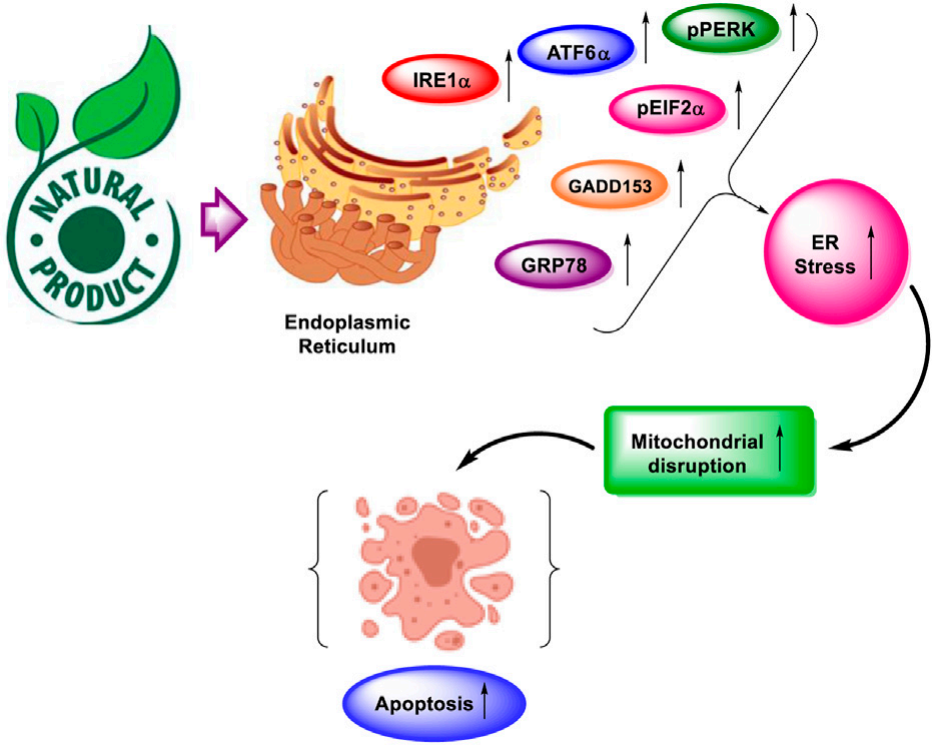
Natural products alleviate endoplasmic reticulum stress for the management of ovarian cancer. Various targets having an association with ER stress and apoptosis are GRP78, GADD153, IRE1α, pPERK, pEIF2α, and ATF6α.

Ovarian cancer spreads to other organs in the vicinity by direct contact. Cancerous cells disassemble from ovarian cancer and metastasize throughout the peritoneum, which affects various organs (Zhang et al., 2019). To reach an efficacious strategy for the treatment of cancer, it is imperative to elucidate the interaction of natural derivatives with cellular targets. As a result, regulating the increase in ER stress-related protein response might be a promising strategy for altering ER homeostasis in cancer cells to induce apoptosis. Several natural products have been reported that activate ER stress-related apoptosis in malignant tumors. These natural products not only induce apoptosis but also lessen the chemotherapy resistance by modulating the ER stress pathways (Limonta et al., 2019). The demonstration of natural products alleviating ER stress for the management of ovarian cancer is described in Figure 1.

Recently, several studies have been carried out to produce novel and innovative anticancer medications by using naturally derived ingredients. Natural products, compared to conventional chemotherapy, seem to have a lower risk for drug resistance and serious adverse effects. For example, fucosterol suppressed the cellular proliferation and progression of the cell cycle in ovarian tumors by influencing the proliferation-related signaling pathways, ER stress, calcium homeostasis, ROS generation, angiogenesis, and mitochondrial functions (Bae et al., 2020a). Fucoidan is a natural product that is derived from brown algae, that is, *Fucus vesiculosus*, *Sargassum fulvellum*, *Undaria pinnatifida*, and *Cladosiphon okamuranus*. Fucoidan lessens the density and quantity of viable ovarian tumor cells, contingent on the types of cell lines (Bae et al., 2020b).

Resveratrol targeted ER stress-mediated apoptosis and downregulated the pathway of hexosamine biosynthesis by disrupting the glycosylation of proteins *via* GSK3β activation. In addition, an ER UDPase, ENTPD5 regulated the glycosylation of proteins by Akt attenuation in ovarian cancerous cells (Gwak et al., 2016). It has been revealed that curcumin is employed in the treatment of ovarian tumor, in which ER stress and ROS (reactive oxygen species) exhibit a significant role in the induction of apoptosis, which suggests that it could be an effective drug in the treatment of ovarian tumor by targeting the oncogenic pathways (Xiao, 2011; Qu et al., 2013).

Quercetin is a flavonoid compound that improves the apoptotic activity of TNF-related apoptosis-inducing ligand (TRAIL) *via* the ROS-mediated CCAAT enhancer-binding homologous protein (CHOP)-death receptor pathway (Yi et al., 2014).

Withaferin-A is a bioactive natural compound isolated from *Withania somnifera* (L.) Dunal. By targeting the putative cancer stem cells, withaferin-A alone and in conjunction with cisplatin reduces the growth and spread of ovarian cancer (Roy et al., 2014). In ovarian cancer, withaferin-A enhances the therapeutic impact of doxorubicin by promoting ROS-mediated autophagy (Zhou et al., 2012). The mechanisms by which withaferin-A accomplishes its cytotoxic potential include the inactivation of NF-kB and Akt to induce apoptosis, lessening the pro-survival of protein Bcl-2, generation of ROS, G2/M cell cycle arrest, DNA damage, activation of caspase 3 and 9 activities, inhibition of HSP90 and Notch-1, induction of Par-4, regulation of FOXO3a, and downregulation of expression of HPV E6 and E7 oncoproteins (Kakar et al., 2012).

The bioactive compound sulforaphane, which is derived from the breakdown of glucoraphanin, has been shown to reduce the proliferation capacity in human (SKOV-3) and mouse (C3 and T3) ovarian cancerous cell lines *via* downregulation of regulators of cell cycle, i.e., cyclin D1 and cyclin-dependent kinase 4 and 6 (cdk4 and cdk6). It constrains the ability of clonogenicity of ovarian tumor cells by the Akt-independent pro-survival pathway such as extracellular signal-regulated kinase/c-Jun-NH2-kinase/p38, which is considered a marker of neoplastic inclination (Chaudhuri et al., 2007). One of the major catechins existing in green tea is epigallocatechin-3-gallate (EGCG). An intensification in p38 mitogen-activated protein kinase (MAPK) activity and a dose-dependent decrease in the matrix metalloproteinase-2 (MMP2) protein level were shown to limit cell the proliferation of OVCAR-3 and migration in human ovarian cancer cells (Wang et al., 2014).

Pulchrin A is a novel naturally derived coumarin derivative of *Enicosanthellum pulchrum* (King) Heusden (synonym of *Disepalum pulchrum* (King) J.Sinclair), which causes apoptosis induction in ovarian cancerous cells (such as CAOV-3 and SKOV-3) *via* intrinsic pathways. It inhibited the G_0_/G_1_ phase of the CAOV-3 cell cycle, causing blebbing of the cell membrane and production of apoptotic bodies, upregulation of Bcl-2-associated X (Bax) protein, downregulation of B-cell lymphoma 2 (Bcl-2), and activation of caspases 3 and 9 (Hsieh et al., 2016). Theaflavin-3,3′-digallate is a black tea polyphenolic compound that is obtained from EGCG polymerization and oxidation. It inhibits tumor angiogenesis more effectively than EGCG. Through the Akt and Notch-1 signaling pathways, theaflavin-3,3′-digallate inhibited the angiogenesis in a human umbilical vein endothelial cell and a chick chorioallantoic membrane model, induced by human ovarian cancer cells (OVCAR-3) (Gao et al., 2016).

Cleistopholine is a natural alkaloid isolated from the roots of *Enicosanthellum pulchrum* (King) Heusden (synonym of *Disepalum pulchrum* (King) J.Sinclair). The cell viability assay was used to determine the cytotoxicity and acridine orange/propidium iodide (AO/PI) assay for the determination of alterations in cellular morphology. Cleistopholine inhibits the proliferation of CAOV-3 cells in ovarian tumor cell lines *via* disruption of the G0/G1 phase in the CAOV-3 cell cycle by increasing the cell cycle arrest continuously, followed by a decrease in the number of cells in the S phase. This clarifies the potential of cleistopholine as a candidate drug to treat ovarian cancer by inducing apoptosis (Nordin et al., 2016). Protoapigenone is a flavonoid compound that is extracted from *Thelypteris torresiana* (Gaudich) Alston (synonym of *Macrothelypteris torresiana* (Gaudich.) Ching plant. The cytotoxic effects of protoapigenone on ovarian cancerous cells were analyzed by FACS and immunoblotting analysis and immunofluorescence investigation by XTT assay. Protoapigenone exhibits a substantial cytotoxic potential on human ovarian cancer cells by inhibition of SKOV-3 and MDAH-2774 cells at the G2/M and S phases of the cell cycle by lowering the expression of p-cyclin B1, cyclin B1, p-Cdk2, and Cdk2 and boosting the inactive p-Cdc25C expressions (Chang et al., 2008).

**FIGURE 2:**
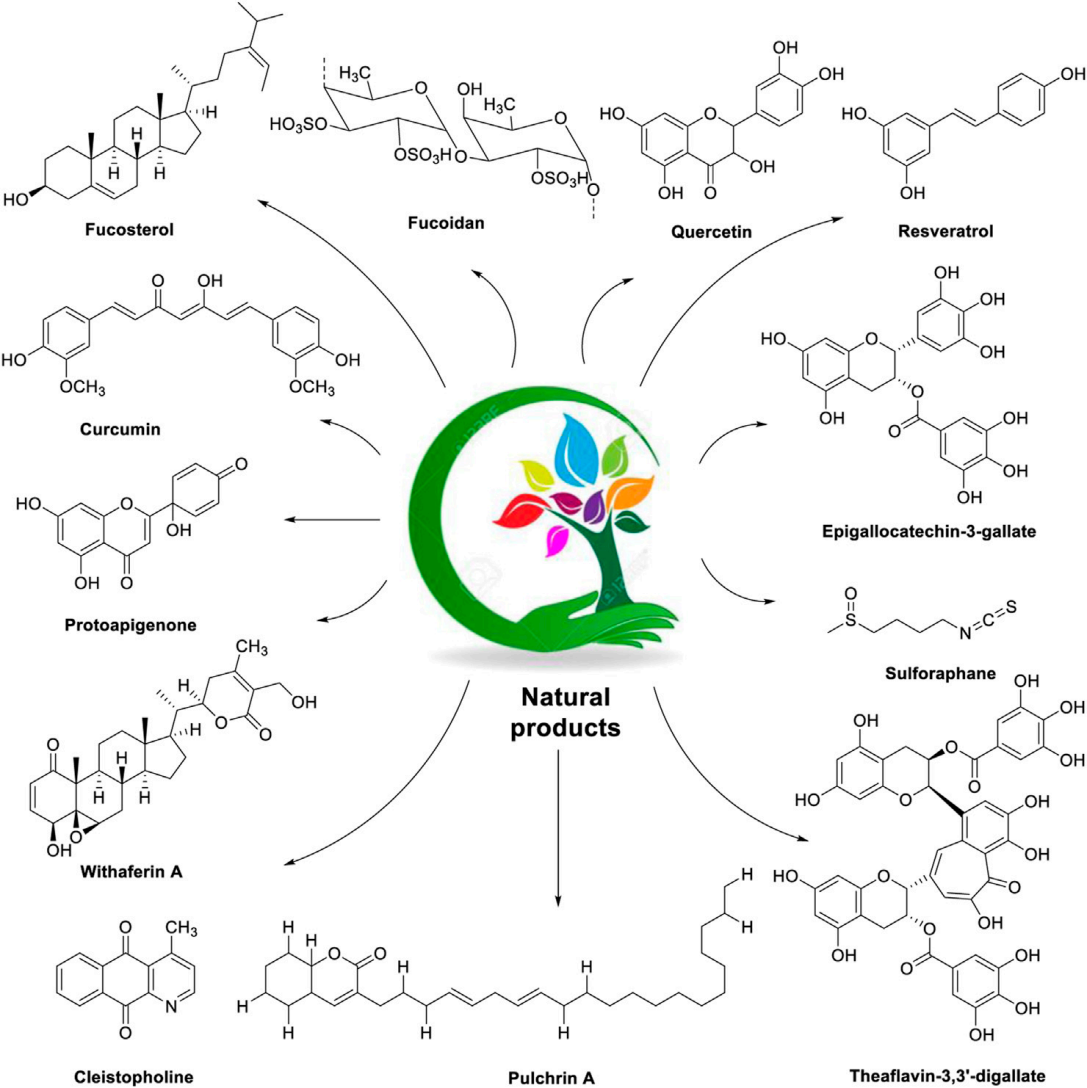
Chemical structures of natural products (fucosterol, fucoidan, quercetin, resveratrol, curcumin, protoapigenone, epigallocatechin-3-gallate, sulforaphane, withaferin-A, cleistopholine, pulchrin A, and theaflavin-3,3′-digallate) used in the management of ovarian cancer with a focus on the ER stress modulator.

The chemical structures of natural products used in the management of ovarian cancer by alleviating endoplasmic reticulum stress are described in Figure 2.

## Nanomaterials and semi-synthetic analogs of natural anti-ER stress agents: approach for the improvement of bioavailability, therapeutic efficacy, and ADMET properties

### Curcumin

Curcumin (**1**) is a natural pigment obtained from *Curcuma longa* L., belonging to the family Zingiberaceae and is widely recognized as a multifunctional compound because of its ample range of pharmacological actions, ranging from antibiotic to anticancer (Shehzad et al., 2010; Heger et al., 2013; Naksuriya et al., 2014; Vallianou et al., 2015). It exhibited numerous activities such as antioxidant, anti-inflammatory, anticancer, and hepatoprotective (Anand et al., 2007; Sreekanth et al., 2011; Khalil et al., 2013). The antitumor property of curcumin is mainly *via* the downregulation of several transcription factors like β-catenin, NF-kB, and AP-1. The clinical application of curcumin has reduced due to its poor solubility, thus confining its bioavailability (Barui et al., 2014; Liu et al., 2014; Nagahama et al., 2015). In order to improve its bioavailability, researchers adopted various strategies like structural modification such as using nanotechnology to develop nanoparticles, niosomes, and liposomes (Kumar et al., 2012; Singh et al., 2015).

**FIGURE 3:**
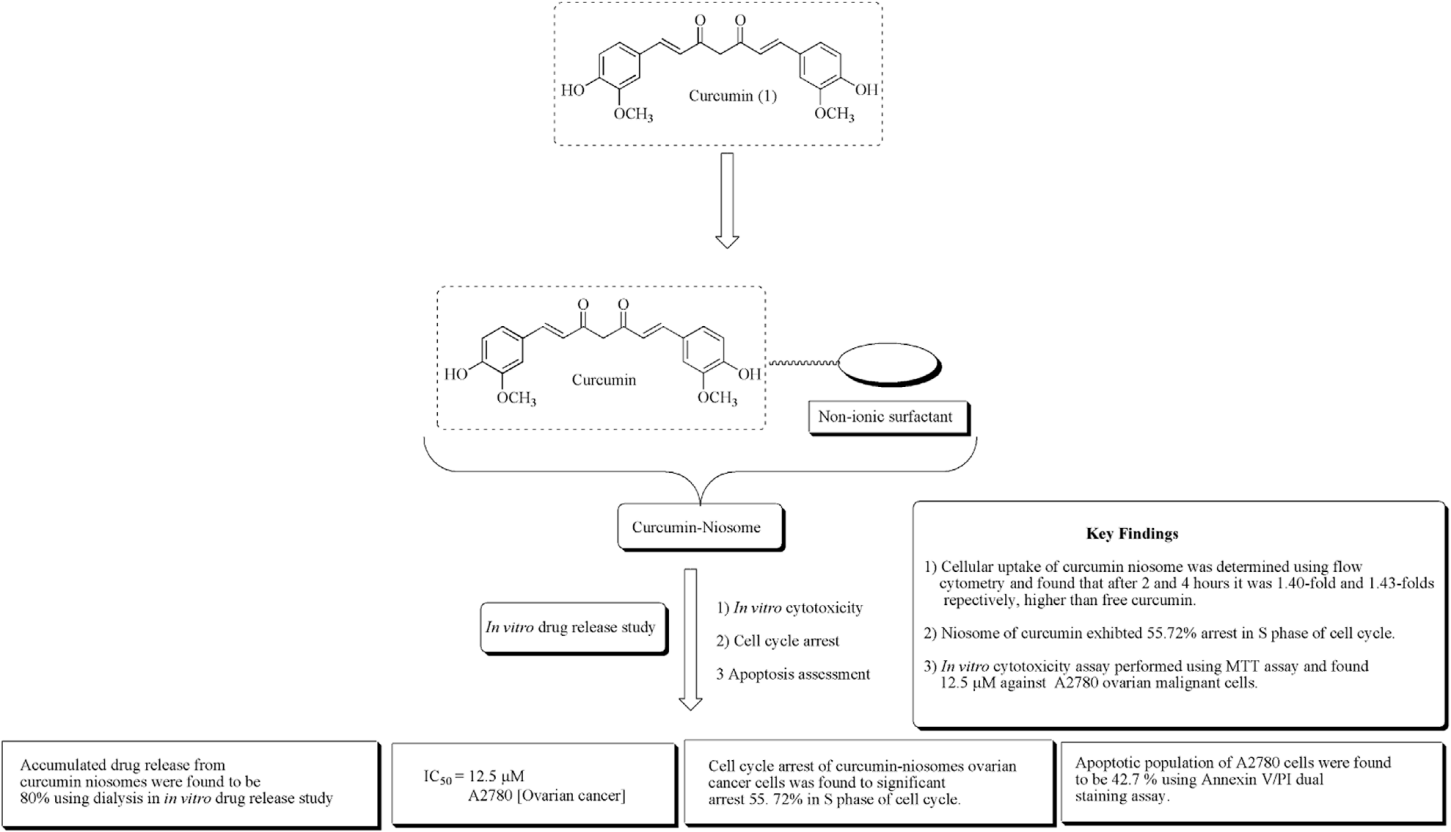
Structure of curcumin along with mechanistic insights of the curcumin niosome system. Curcumin niosomes were found to have a significant arrest in the S phase of the cell cycle.

Ying et al. established novel niosomes of curcumin using non-ionic surfactant in a mixture of tween 80, span 80, and poloxamer 188 in the ratio of 3:1:1. The developed niosomes of curcumin subjected to *in vitro* cytotoxicity against human ovarian cancerous A2780 cell lines and *in vitro* drug release (Xu et al., 2016). The structure of curcumin along with its mechanistic insights is depicted in Figure 3.

**FIGURE 4:**
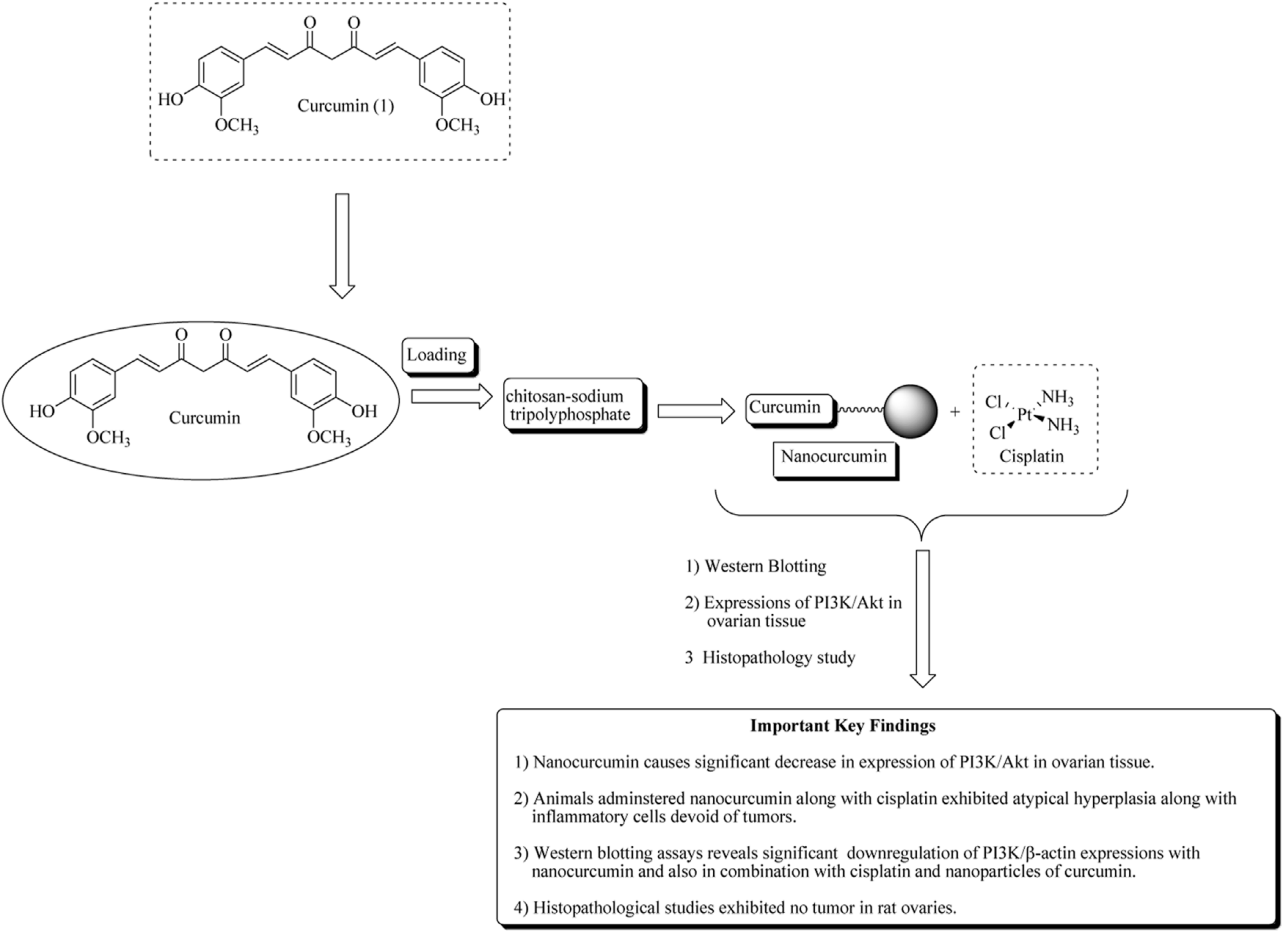
Mechanistic insights of curcumin nanoparticles along with important key findings. Curcumin nanoparticles or nanocurcumin loaded on chitosan–sodium tripolyphosphate significantly decreases PI3K/Akt expression in ovarian tissue.

In another approach, Sandhiutami et al. reported the evaluation of curcumin nanoparticles against ovarian cancer. The main aim of the study is to improve the bioavailability of curcumin. The author developed the nanoparticles by loading curcumin in a chitosan–sodium tripolyphosphate system. Characterization of nanocurcumin was determined using particle size, entrapment efficiency, and zeta potential. Nanocurcumin was found to increase 20-fold plasma concentrations than free curcumin. Nanocurcumin also enhances the anticancer actions of cisplatin against ovarian cancer in rats by reducing the expressions of PI3K, JAK, STAT3, and Akt phosphorylation (Sandhiutami et al., 2021). Mechanistic insights of curcumin nanoparticles are presented in Figure 4 along with important key findings.

**FIGURE 5:**
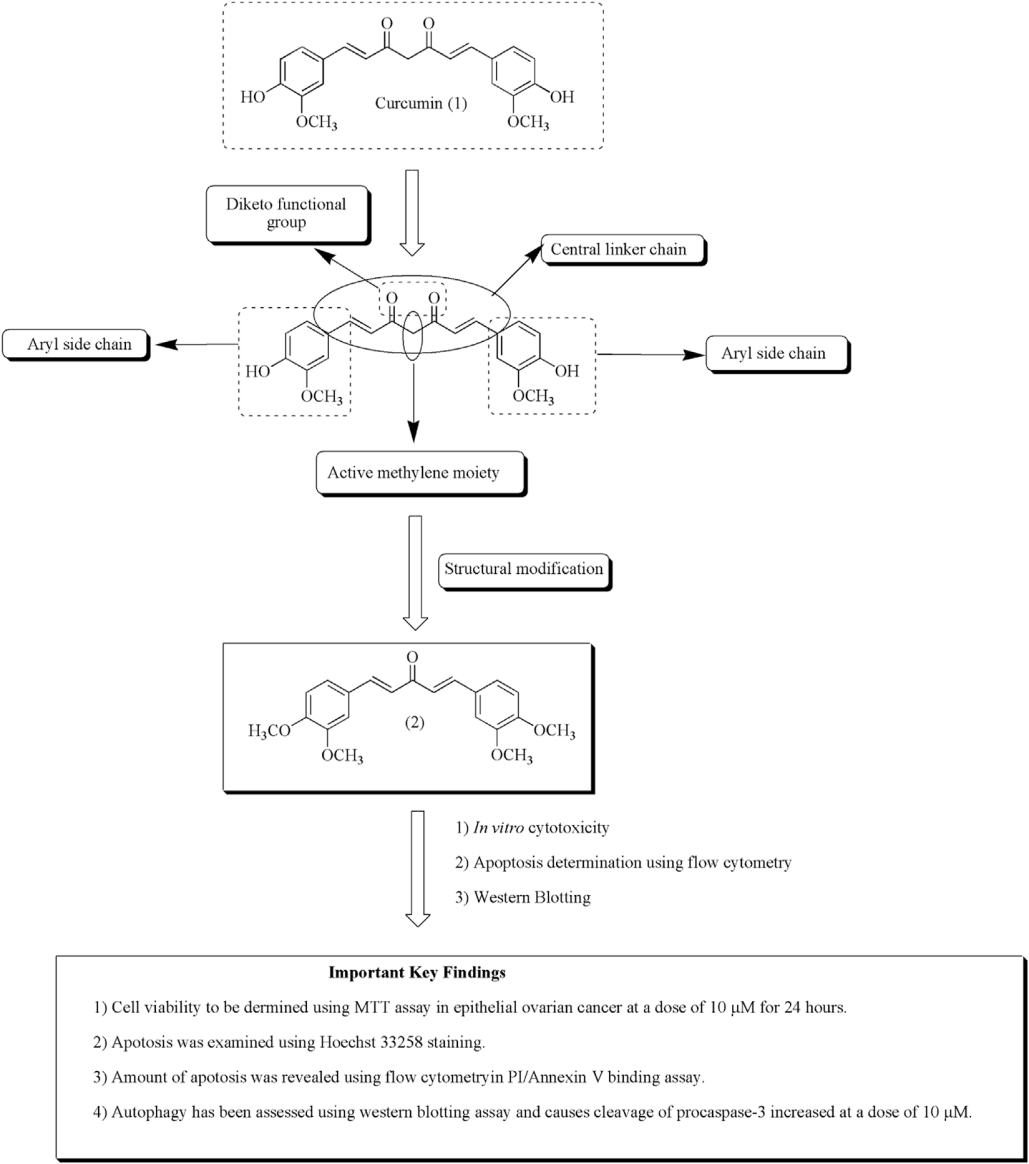
Structure–activity relationships of curcumin along with mechanistic insights. Anti-ovarian cancer potential of curcumin analog (2) was validated through MTT assay, Hoechst staining, flow cytometry, and Western blotting assay.

Wanglei et al. reported apoptosis caused by the curcumin analog in epithelial ovarian cancer *via* targeting autophagy and ER stress signaling pathway and hence become a novel target for the mitigation of ovarian tumor. Compound (2) also persuades autophagy by 3-methyladenine in HO8910 cells (Qu et al., 2013). Figure 5 depicted the mechanistic insights of compound (**2**) along with the structure–activity relationships of curcumin.

### Quercetin

**FIGURE 6:**
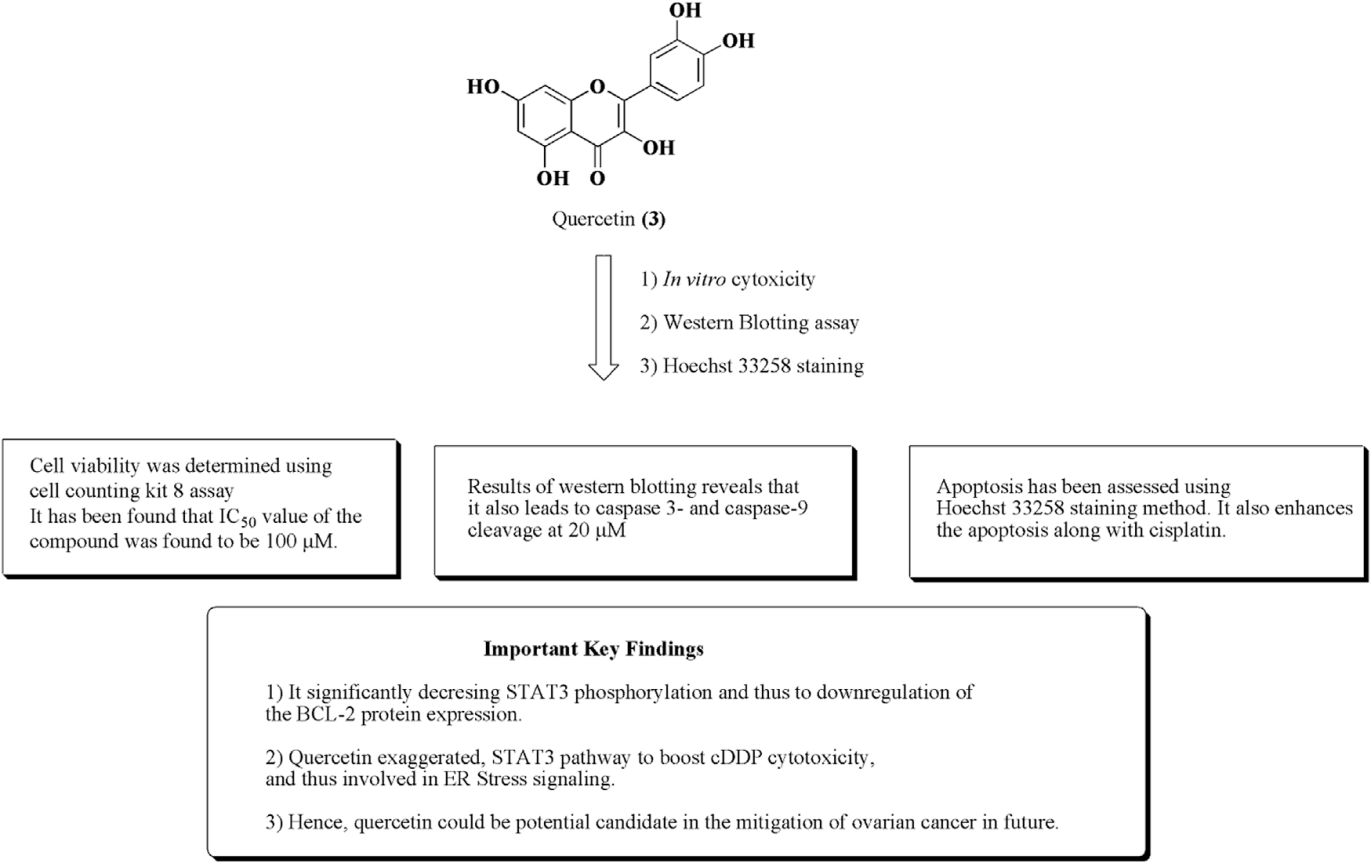
Structure of quercetin along with important key findings. Anti-ovarian cancer potential of quercetin was assessed by *in vitro* cytotoxicity, Western blotting assay, and Hoechst 33258 staining.

Quercetin (**3**) is a polyphenolic flavonoid-based compound obtained from citrus fruits, onions, and grapes (Xu D et al., 2019; Sharma et al., 2021). Numerous reports established its anticancer potential against various human cancer cell lines such as ovarian cancer, breast cancer, and prostate cancer. It also demonstrated to have an ample range of activities such as antioxidant, anti-inflammatory, antiviral, CNS, and CVS disorders (Miean and Mohamed, 2001; Somerset and Johannot, 2008; Jain et al., 2013; Kumar et al., 2015; Kumar et al., 2016a; Kumar et al., 2016b; Kumar et al., 2018a; Kumar et al., 2018b; Kaur et al., 2020; Kumar et al., 2021). Quercetin also potentiates the cytotoxicity of cisplatin in ovarian cancer via activation of ER stress and suppression of STAT phosphorylation, and hence induction of apoptosis has been presented in Figure 6.

**FIGURE 7:**
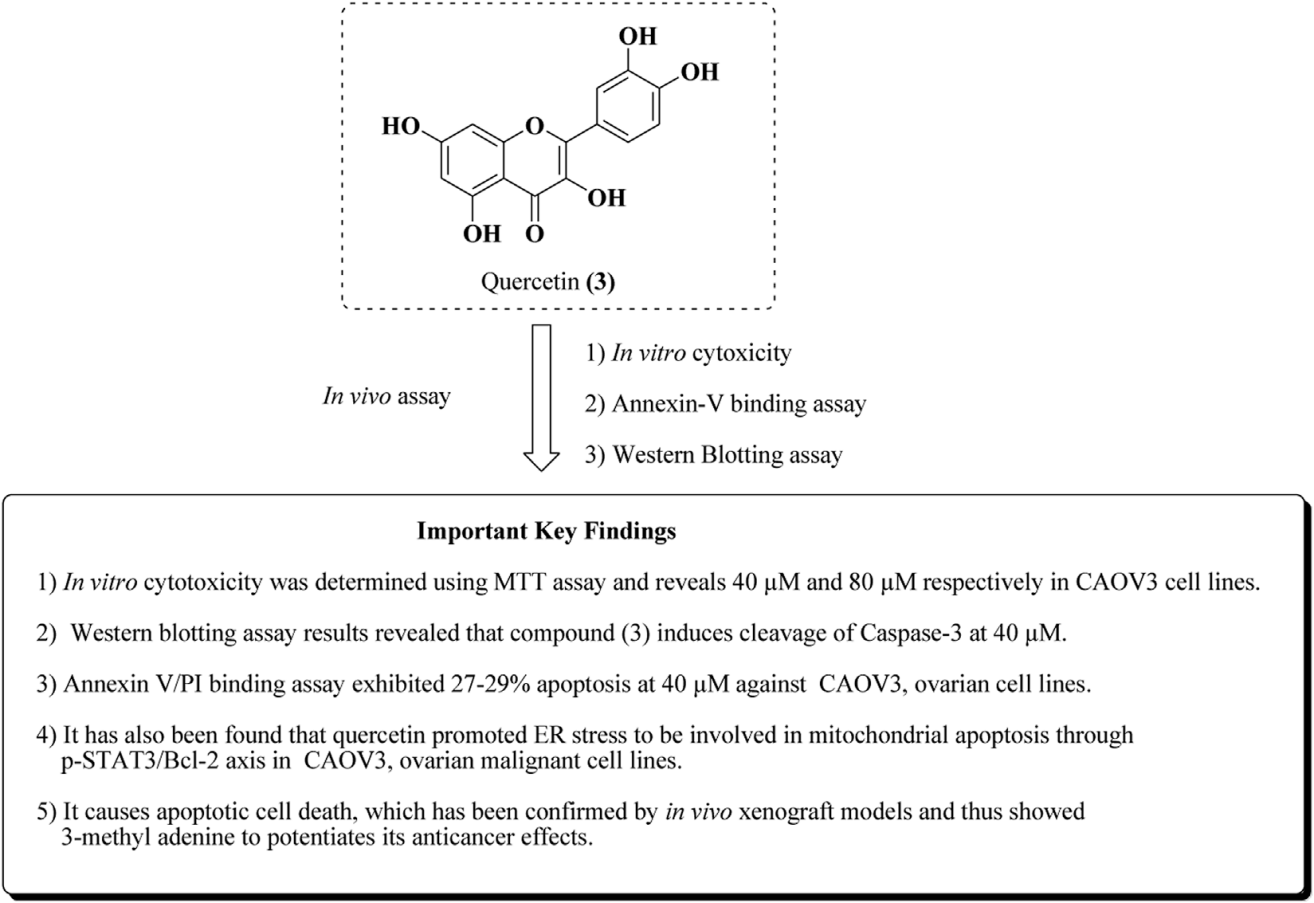
Mechanistic insights of quercetin by *in vitro* and *in vivo* experiments. Quercetin was assayed for anti-ovarian cancer activity in CAOV-3 cell lines and the *in vivo* xenograft model.

Similarly, in addition to this, Liu et al. reported the apoptotic effect of quercetin in ovarian cancer by ER stress *via* the p-STAT3/Bcl-2 axis (Liu et al., 2017). The results of *in vitro* and *in vivo* studies are presented in Figure 7.

**FIGURE 8:**
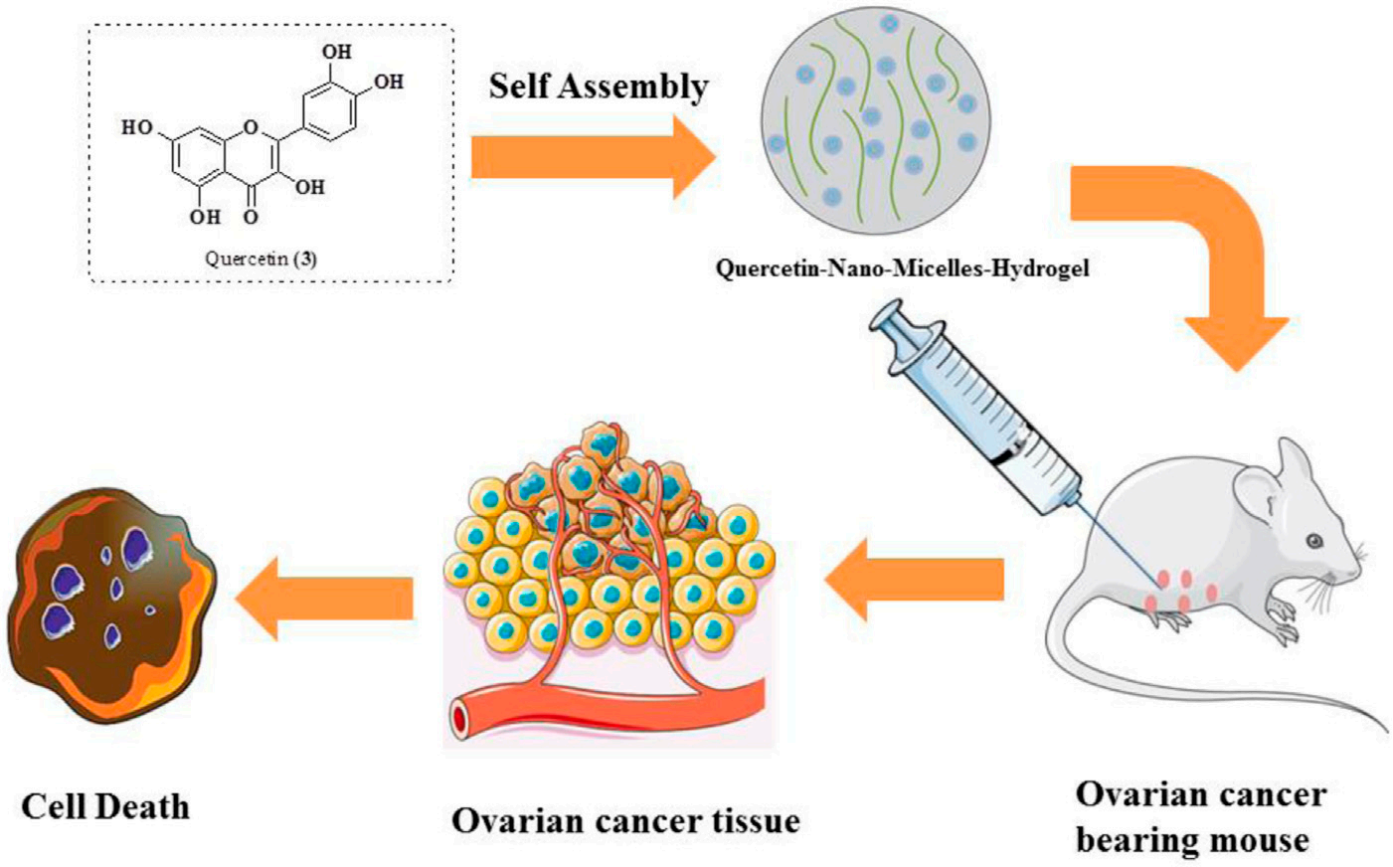
Anticancer effects of quercetin–micelle–hydrogels on an ovarian cancer-bearing mouse.

Xu et al. studied the anticancer potential of self-assembly micelles hydrogel of quercetin against ovarian cancer. After administration of quercetin hydrogel through the parenteral route, it was found that the bioavailability of quercetin hydrogel has been improved. Quercetin was encapsulated in MPEG-PCL and then suspended in a hydrogel. The quercetin–micelle–hydrogel was injected into the ovarian cancer-bearing mouse. The results reveal that there is a slow release of quercetin near the tumor and thus be much more effective in the mitigation of ovarian cancer using *in vivo* models (Figure 8). The quercetin-micelles-hydrogel also exhibited apoptotic effects using *in vitro* cytotoxicity toward SKOV-3 cell lines in MTT assay and annexin-V/PI binding assay (Xu et al., 2018).

### Morusin

**FIGURE 9:**
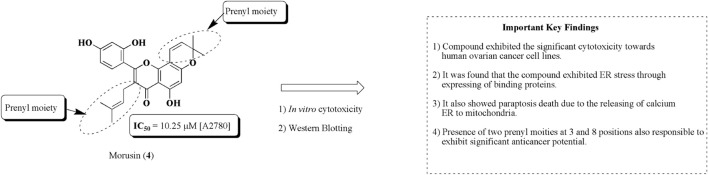
Structure of morusin along with important key findings. Anti-ovarian cancer potential of morusin was assessed by *in vitro* cytotoxicity and Western blotting. Prenyl moieties seem to be structurally responsible for this activity.

Morusin (4) is a flavonoid-based compound obtained from the bark of *Morus australis* Poir. belonging to the family Moraceae. The literature reveals that the compound has been recognized to exhibit significant antioxidant, anti-inflammatory, antibacterial, and anticancer properties toward human cancerous cell lines (Martucciello et al., 2020). Xue et al. (2018) established the potential of morusin (4) against ovarian cancer. The results revealed that the compound has the capacity to produce ER stress. It has also been found that dilation of mitochondria and ER was observed owing to the release of calcium from the endoplasmic reticulum to mitochondria. The structure of morusin and its important key findings are presented in Figure 9.

### Epicatechin gallate

**FIGURE 10:**
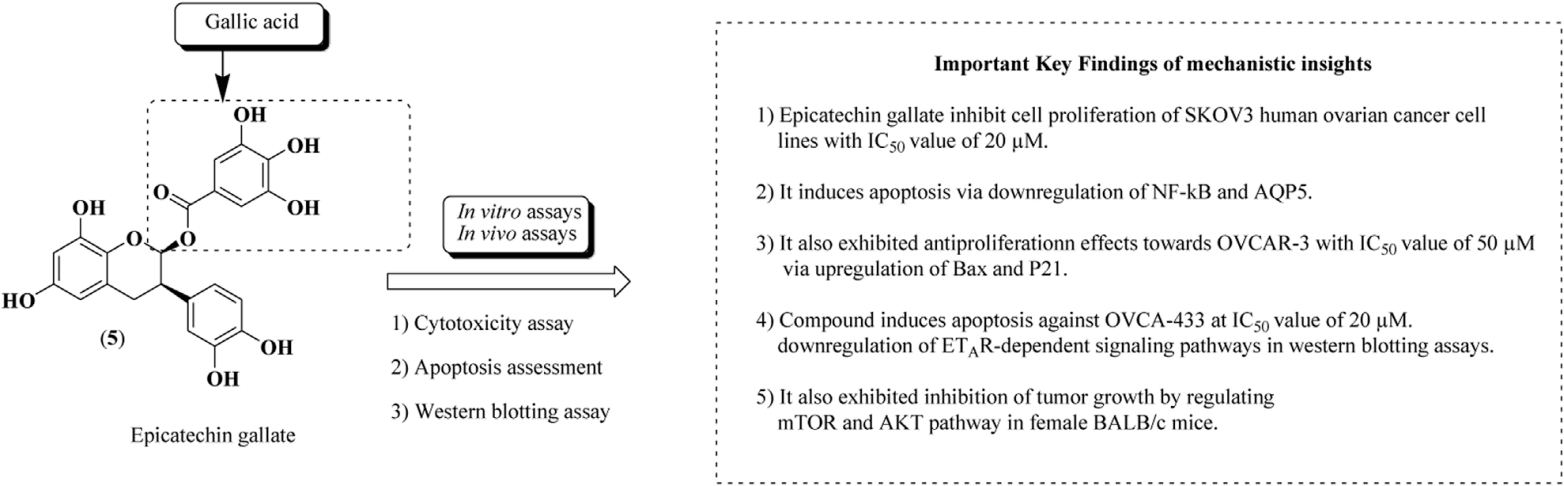
Structure of epicatechin gallate along with mechanistic insights. Epicatechin gallate was subjected to both *in vitro* and *in vivo* assays to validate the anti-ovarian cancer potential. SKOV-3, OVCAR-3, and OVCA-433 cell lines were used.

Epicatechin gallate (5) is a natural bioactive flavonoid obtained from grapes (*Vitis sp.*) and green tea (*Camellia sinensis* (L.) Kuntze). Epicatechin-3-O-gallate is a gallate ester attained by condensation of the carboxy moiety of gallic acid with the introduction of the (3R)-hydroxy group of epicatechin. Epicatechin gallate is recognized for its ample biological activities, viz, anticancer against human ovarian and breast cancer cell lines, antioxidant, anti-inflammatory, and antimicrobial properties (Sharma et al., 2021). Numerous nanoformulations of epicatechin gallate and epicatechin digallate have been prepared and established their anticancer potential against ovarian, prostate, colon, and breast cancers. PLGA nanoparticles and solid lipid nanoparticles of epicatechin digallate of green tea have been reported for their antiproliferative effects using *in vitro* and *in vivo* models (Jiang et al., 2021). Epicatechin gallate had been evaluated against ovarian cancer cell lines such as SKOV-3, OVCAR-3, OVCA-433, and HEY human ovarian cancer cell lines using *in vitro* cytotoxicity studies (Huh et al., 2004; Spinella et al., 2006; Chen et al., 2011; Singh et al., 2011; Yan et al., 2011; Huang et al., 2020; Qin et al., 2020). The structure of epicatechin gallate along with mechanistic insights for anticancer potential is represented in Figure 10.

### Evodiamine

**FIGURE 11:**
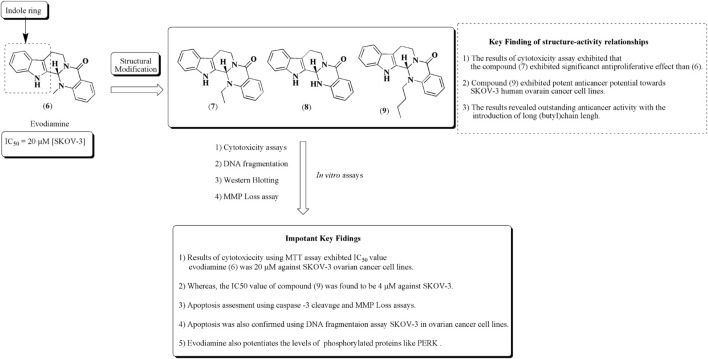
Structure of evodiamine along with structure–activity relationships. Anti-ovarian cancer potential of evodiamine was assessed by cytotoxicity assay, DNA fragmentation, Western blotting, and MMP loss assay. The structural analogs of evodiamine are 7, 8, and 9.

Chen et al. established the apoptotic effect of evodiamine (6) isolated from the bark of *Evodia ruticarpa* (A. Juss.) (Hook.f. and Thomson (synonym of *Tetradium ruticarpum* (A. Juss.) T.G.Hartley)), belonging to the family Rutaceae. It is an indole-based alkaloid. Evodiamine is also known to have a wide range of pharmacological properties, such as anticancer, vasorelaxant, and anti-nociceptive actions. The bioavailability problem of evodiamine has been resolved by structural modification and by using nanotechnology, which may also increase its absorption. Zou et al. (2014) reported the preparation of PLGA-loaded nanoparticles of evodiamine. Similarly, another strategy has been used to prepare nanoparticles of evodiamine targeting EGFR for the mitigation of ovarian, breast, and colorectal cancers (Li et al., 2019). Results of cytotoxicity studies reveal that introduction of the butyl group presented in compound (9) exhibited significant apoptotic effects and increased expression of PERK against SKOV-3 human ovarian cancer cell lines (Chen et al., 2016). The structure–activity relationships along with important key findings are represented in Figure 11.

### Fucosterol

**FIGURE 12:**
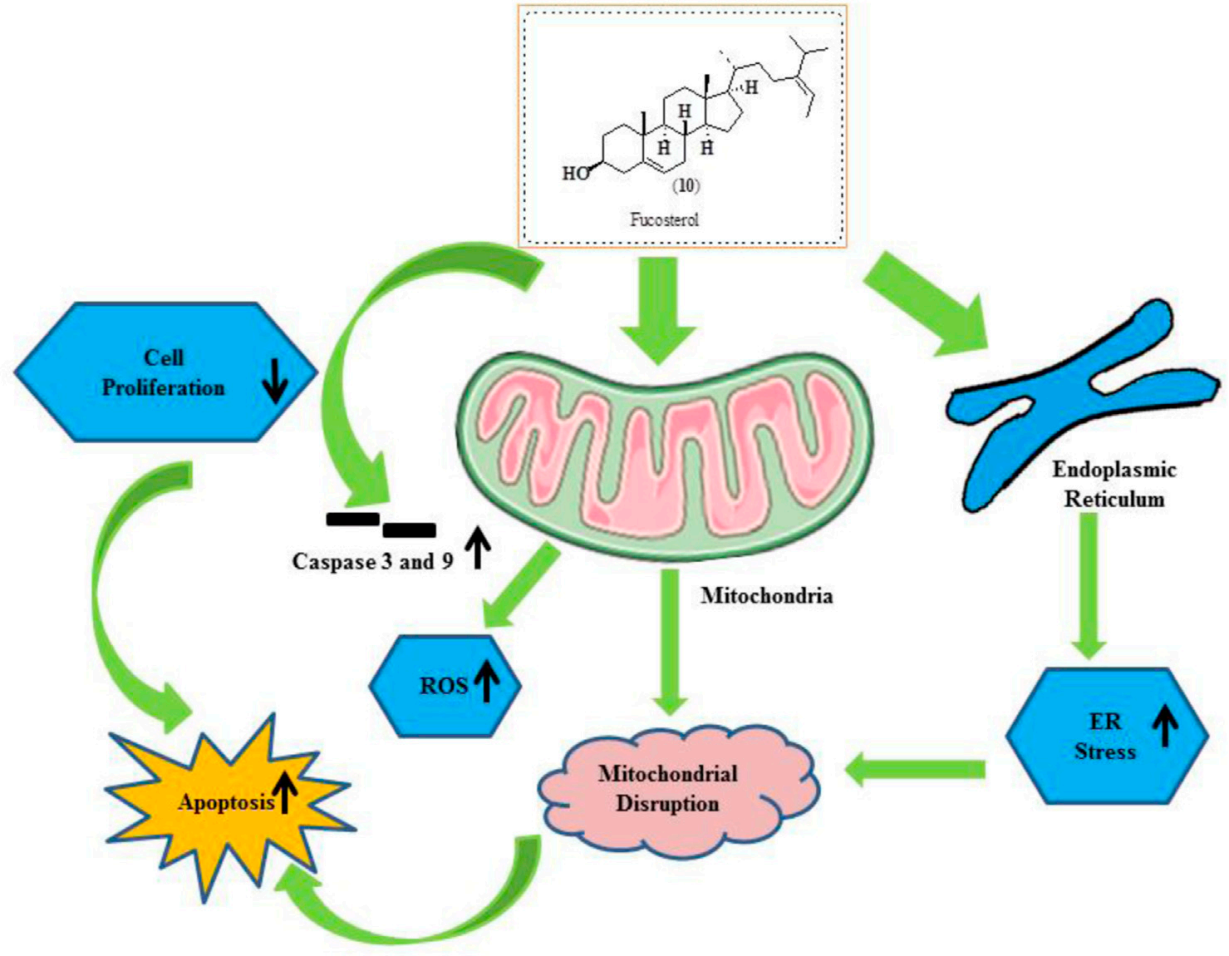
Structure of fucosterol along with its mechanistic insights. Fucosterol was found to modulate caspase-3 and caspase-9, ROS, mitochondrial disruption, ER stress, and apoptosis.

Fucosterol (10) is phytosterol extracted from algae and seaweed. It is recognized to have several therapeutic actions like an antiepileptic, antidepressant, and anticancer effects against lung, colon, breast, and cervical cancers (Sheu et al., 1998; Sheu et al., 2007; Ostad et al., 2012; Jiang et al., 2018; Pacheco et al., 2018). Rajeshkumar et al. (2013). reported the gold-plated NPs of fucosterol extracted from *Turbinaria conoides*. Furthermore, Bae et al. (2020)a. reported the effects of fucosterol against ovarian cancer *via* inducing ER stress and mitochondrial dysfunctions. It also exhibited cell cycle arrest in OV90 cells. Compound (10) also reduces the tumor progression in zebrafish using an *in vivo* xenograft model. The structure of fucosterol along with its mechanistic insights is presented in Figure 12.

### Paclitaxel

**FIGURE 13:**
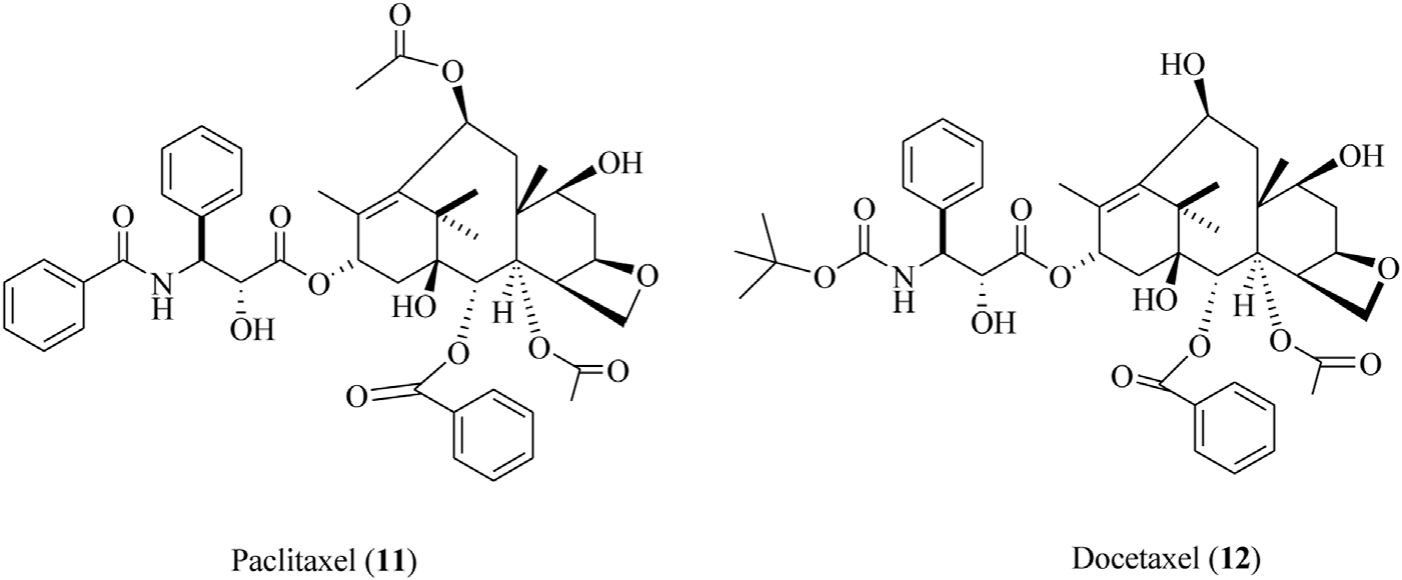
Structures of paclitaxel and its semi-synthetic derivative, docetaxel.

Paclitaxel (11) is also renowned as a taxol that is isolated from the bark of *Taxus brevifolia* Nutt., commonly known as the Pacific yew tree (Kumar and Kumar Jain, 2016; Kumar et al., 2017). The semi-synthetic derivative of paclitaxel is also known as docetaxel (12). Both these compounds (11, 12) were clinically approved for the cure and mitigation of lung, prostate, breast, and ovarian cancers (Downing and Nogales, 1998; Schmidt and Bastians, 2007). The structures of paclitaxel and docetaxel are depicted in Figure 13.

**FIGURE 14:**
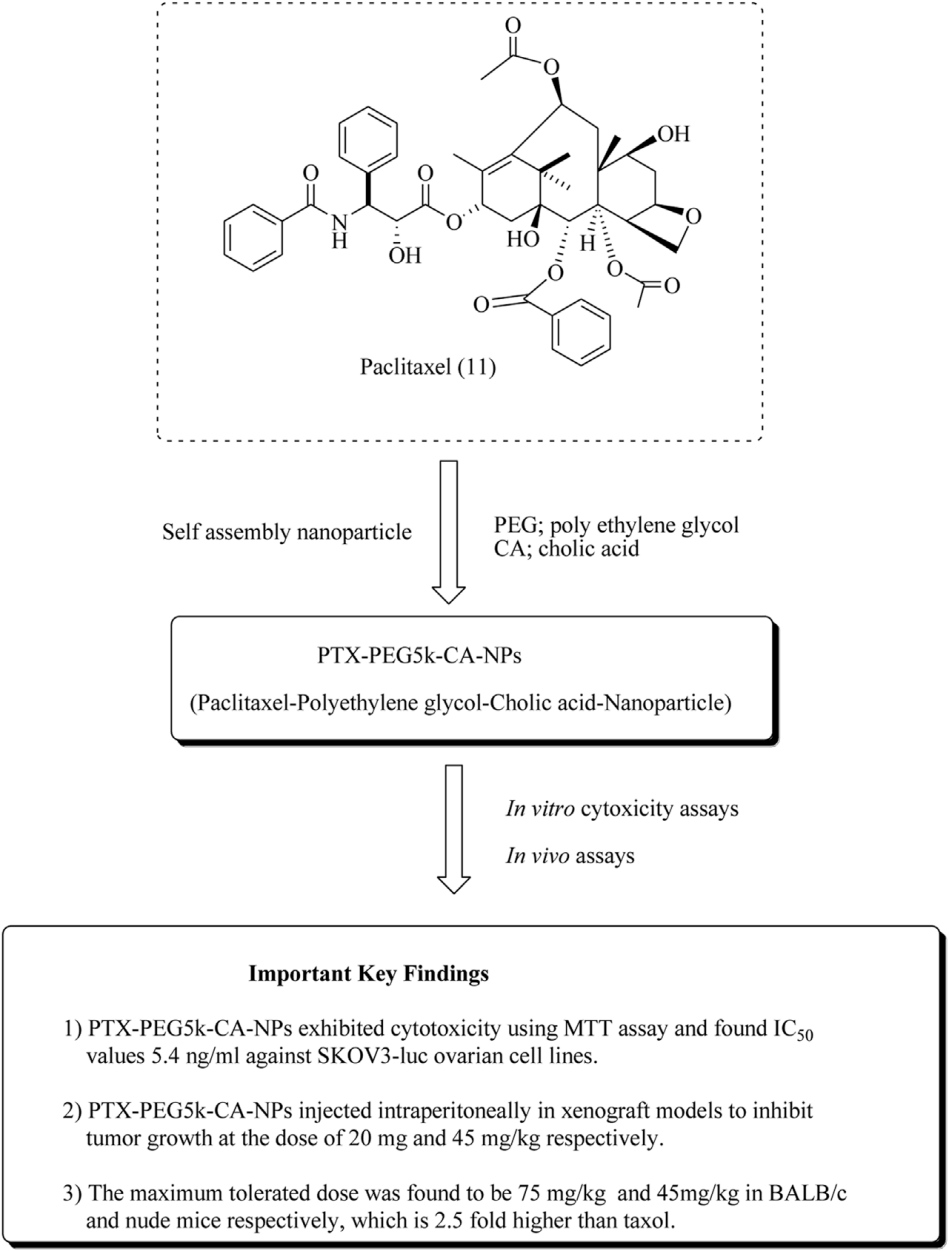
Anticancer potential of paclitaxel nanoparticles against ovarian cancer. PTX-PEG5k-CA-NPs were assessed for anti-ovarian cancer potential using *in vitro* and *in vivo* assays. SKOV-3-Luc cell lines and BALB/c and nude mice were used.

Xiao et al. (2009)reported the synthesis and anticancer potential of paclitaxel nanoparticles (PTX-PEG5k-CA-NPs; paclitaxel–polyethylene glycol-cholic acid nanoparticles) toward ovarian cancer. The results of both *in vitro* and *in vivo* studies are presented in Figure 14, along with their maximum tolerated dose.

**FIGURE 15:**
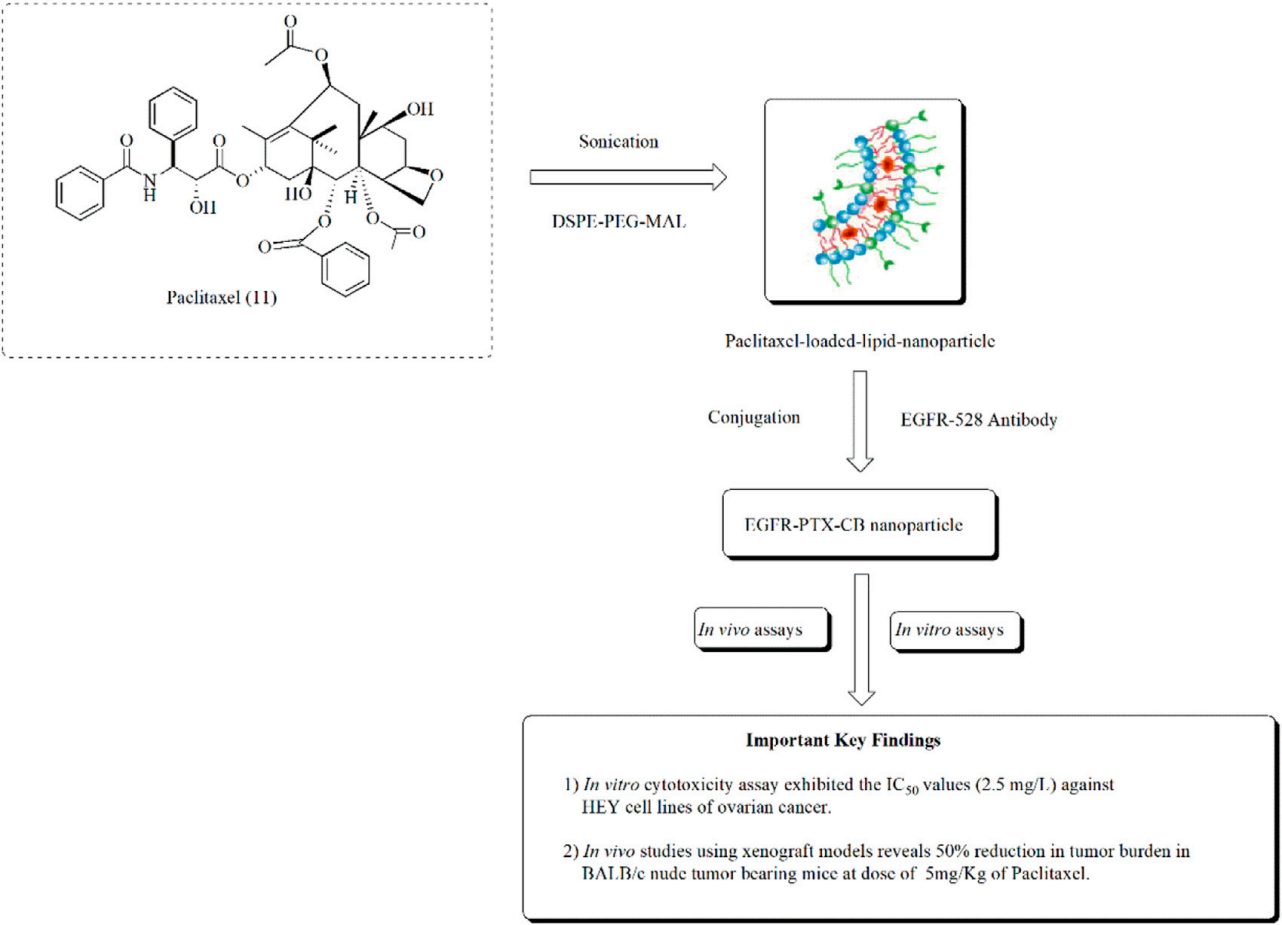
Ameliorating effect of paclitaxel-loaded EGFR-conjugated nanoparticles against ovarian cancer. *In vitro* assays using HEY cell lines and *in vivo* assays using BALB/c nude tumor-bearing mice were performed to assess the anti-ovarian cancer potential of EGFR-PTX-CB nanoparticles.

Similarly in another approach, Zhai et al. (2018). studied the amelioration of ovarian cancer using lipid nanoparticles of paclitaxel. Paclitaxel-loaded lipid nanoparticles were prepared using sonication. These nanoparticles exhibited significant anticancer effects in HEY ovarian cancer cell lines at the IC_50_ value of 2.5 mg/L. The anticancer effect of paclitaxel lipid nanoparticles is presented in Figure 15.

### Resveratrol

**FIGURE 16:**
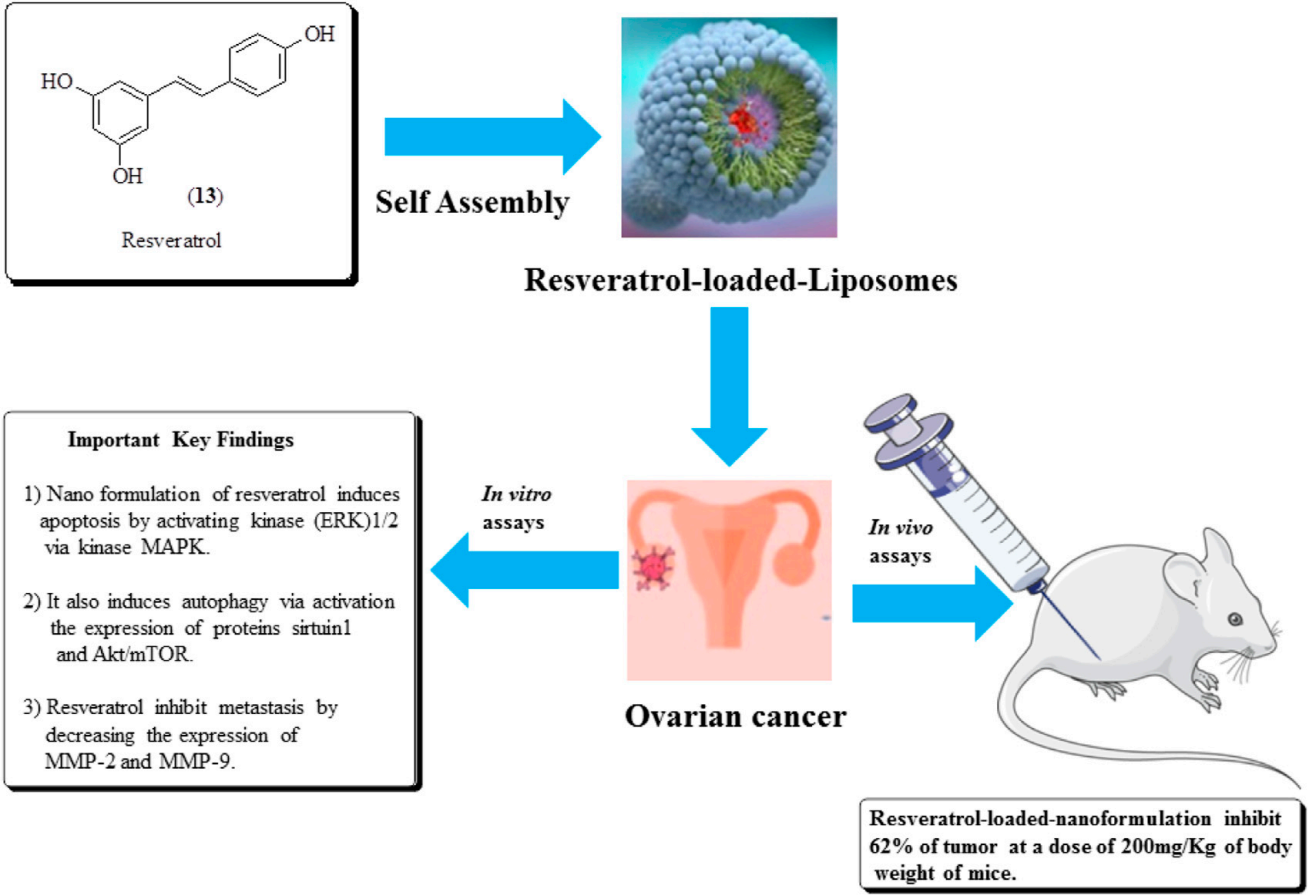
Structure of resveratrol and its anticancer potential along with its mechanistic insights. Resveratrol-loaded liposomes were assessed for ovarian cancer treatment using *in vitro* and *in vivo* assays.

Opipari et al. (2004) established the anticancer potential of resveratrol (13) against ovarian cancer and found that it induces autophagy in ovarian cancer cells. Similarly, in another study, Guo et al. (2010) reported the antitumor effects of the nano-formulation of resveratrol against ovarian cancer in nude mice. It causes a 62% reduction in the tumor size at a dose of 200 mg/kg of the body weight of mice. The structure of resveratrol and its anticancer potential is presented in Figure 16 (Sharifi-Rad et al., 2021).

### Sulforaphane

**FIGURE 17:**
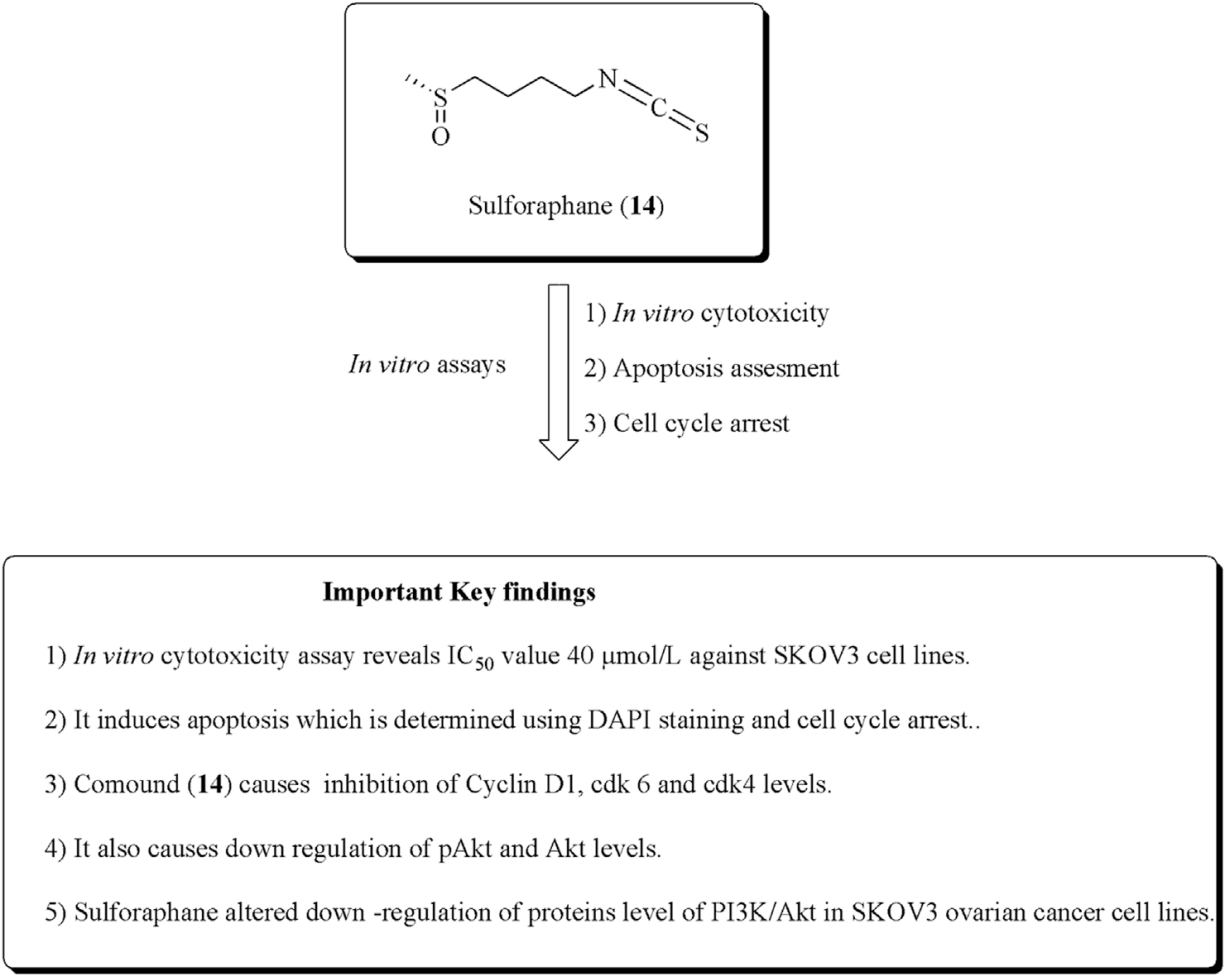
Structure of sulforaphane along with its mechanistic insights. Sulforaphane was found to exert cytotoxic and apoptotic effects against SKOV-3 cell lines and modulates the cyclin D1, cdk6, cdk4, pAkt, and Akt levels.

Sulforaphane (14) is the main constituent of cruciferous vegetables, especially broccoli, Brussels sprouts, and cauliflower. It has the ability to inhibit proliferation in ovarian and prostate cancer cell lines (Zhang et al., 1992; Zhang et al., 1994; Chinni et al., 2001; Murillo and Mehta, 2001; Jung Park and Pezzuto, 2002; Naora and Montell, 2005; W. Watson et al., 2013). Chaudhuri et al. (2007) established the potent antiproliferative effects of sulforaphane against SKOV-3 ovarian human malignant cell lines along with IC_50_ values of 40 μmol/L. It also induces apoptosis and inhibition of cyclin D1, ckd6, and ckd4 levels. Similarly, in another approach, Xu Y et al. (2019). established the anticancer potential of sulforaphane–cisplatin nanoparticles and reduced the side effects of cisplatin by decreasing GSH levels. The structure of compound (14) along with its mechanistic insights is depicted in Figure 17.

**FIGURE 18:**
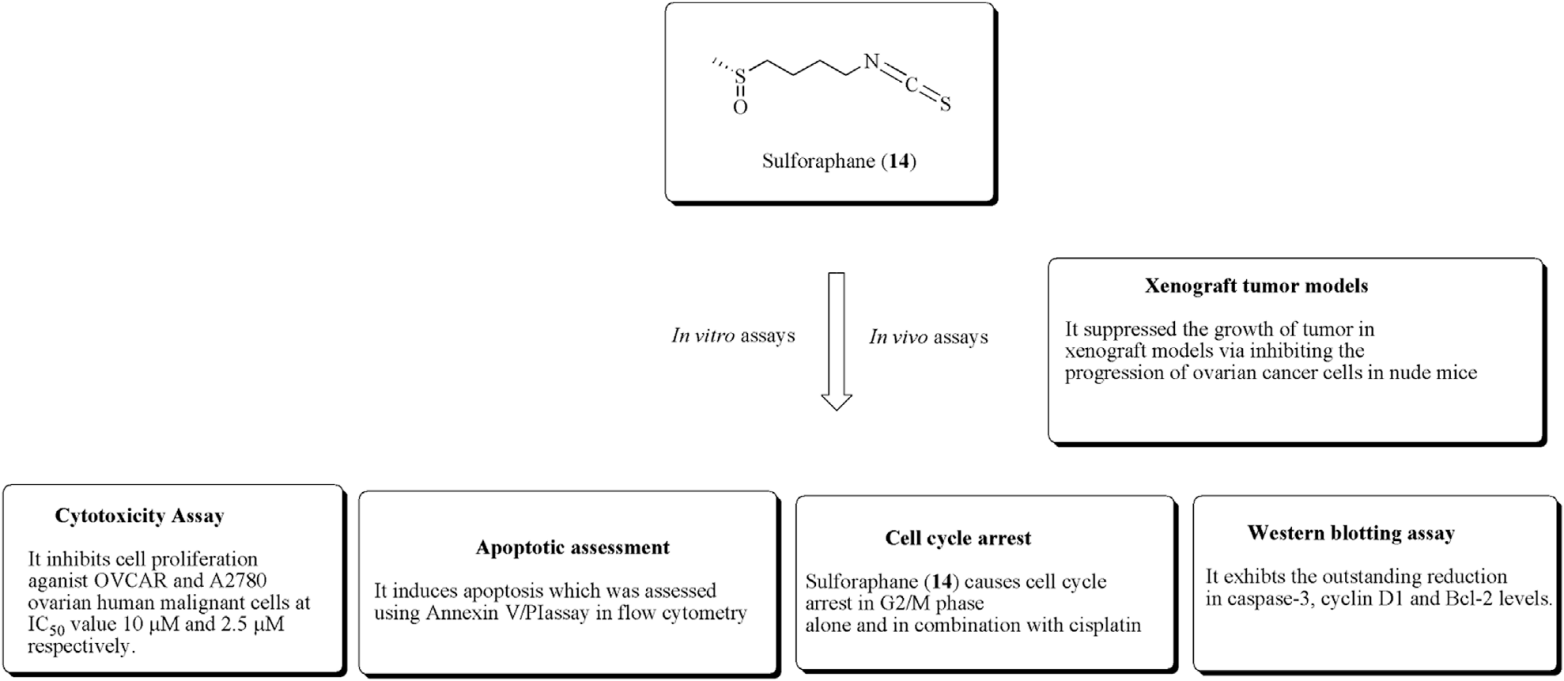
Antiproliferative effects of sulforaphane using *in vitro* and *in vivo* experiments. OVCAR and A2780 ovarian cell lines along with xenograft tumor models were used for this activity validation.

In another study, Kan et al. reported the antiproliferative effects of sulforaphane toward OVCAR and A2780 ovarian human malignant cells at IC_50_ values of 10 and 2.5 µM, respectively. It also reduces the suppression of tumor growth *via* the reduction of proliferation in xenograft models using nude mice (Kan et al., 2018). The anticancer potential of compound (14) using *in vitro* and *in vivo* experiments is presented in Figure 18.

### Theaflavin-3,3ʹ-digallate

**FIGURE 19:**
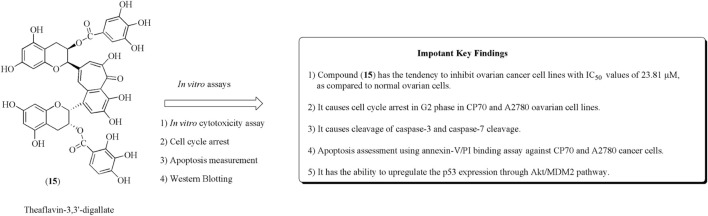
Structure of compound (15) along with its mechanistic insights. CP70 and A2780 cell lines were used.

Theaflavin-3,3ʹ-digallate (15) is a polyphenol obtained from black tea. Compound (15) has anticancer potential against CP70 and A2780 ovarian cancer cell lines (McLean et al., 2009; Pan et al., 2018). It is generally used in cisplatin-resistant ovarian cancer. Jiang et al. reported the PLGA-loaded nanoparticles of catechins including theaflavin-3,3ʹ-digallate and epicatechin-3,3ʹ-digallate and evaluated their anticancer potential against ovarian and breast cancer (Jiang et al., 2021). Youying et al. established that it has an apoptotic effect on ovarian cancer cell lines and causes arrest in the G2 phase *via* upregulating p53 protein expression (Tu et al., 2016). The structure of theaflavin-3,3ʹ-digallate along with its anticancer effect is presented in Figure 19.

### Miscellaneous

**FIGURE 20:**
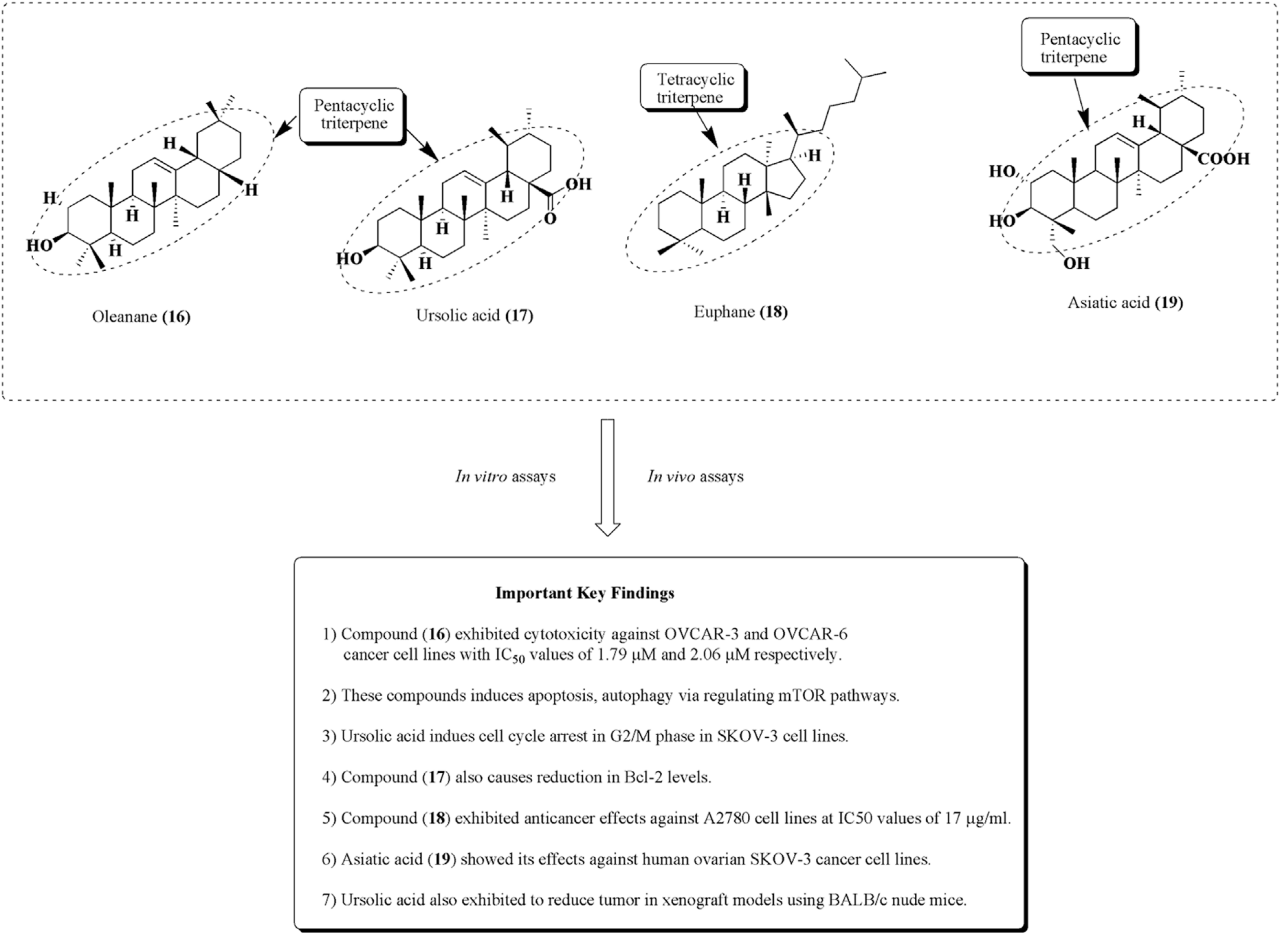
Structures of some triterpenes as potential anticancer agents against ovarian cell lines. Anti-ovarian cancer activity of these tetracyclic and pentacyclic triterpenes was assessed by performing *in vitro* and *in vivo* assays.

Anna Kaps et al. reported the nanoformulations of triterpenoids such as ursolic acid, betulinic acid, and oleanane along with their effective use in the mitigation of diverse types of cancer such as breast, ovarian, lung, and prostate cancers. The main objective is to formulate the nanocarriers of these bioactive molecules to enhance their transport ability, including solubility bioavailability, and reduce their toxicity and side effects (Kaps et al., 2021). An oleanane (16) is a triterpenoid saponin isolated from *Bolbostemma paniculatum* (Maxim.) Franquet is documented for its anticancer potential against ovarian cancer cell lines such as OVCAR-3 and OVCAR-6, with IC_50_ values of 1.79 and 2.06 µM, respectively (Gao et al., 2013; Zafar et al., 2018; Şoica et al., 2021). Ursolic acid (17) is another pentacyclic triterpene, established as an anticancer agent against ovarian cancer cell lines such as SKOV-3. It induces apoptosis and cell cycle arrest in the G2/M phase *via* the reduction in Bcl-2 levels and increased ROS production (Lin and Ye, 2020). It is also used along with cisplatin and exhibits an outstanding reduction in the ovarian tumor size in the xenograft model using BALB/c nude mice. Euphane (18) has also been reputable for its effects on A2780 ovarian cancer cells, with an IC_50_ value of 17 μg/ml (Hou et al., 2010; Cheng et al., 2013). Similarly, in another study, asiatic acid (19) was isolated from *Centella sp*, and it is recognized for its antitumor effects against ovarian metastatic SKOV-3 malignant cell lines (Jia et al., 2017; Wang X et al., 2017). The structures of potential triterpenes along with their mechanistic insights against human ovarian cancer cell lines are presented in Figure 20. Summary of some phytomolecules and their nanosystems along with their *in vitro* and *in vivo* evaluation against various human ovarian cancer cell lines using different cancer models and their outcomes presented in Table 1.

**TABLE 1:** Summary of some anti-ovarian phytomolecules and their nanosystems.

S. No.	Name of natural anti-ER stress agents	Nanomaterial	Cancer model	Outcome/Result	Reference
1	Curcumin	Noisome	*In vitro* cytotoxicity against ovarian A2780 cell lines	IC_50_ values of 12.5 µM	Xu et al. (2016)
			Cell cycle arrest	55.72% cell cycle arrest in the S phase	
			Apoptosis assessment	42.7% using annexin-V/PI dual staining assay	
2	Curcumin	Nanoparticles (chitosan–sodium tripolyphosphate system)	Western blotting	Reducing proteins expressions of PI3K, JAK, STAT3, and Akt phosphorylation	Sandhiutami et al. (2021)
3	Quercetin	Micelle–hydrogel– quercetin	*In vitro* cytotoxicity assay	Reduction of growth inhibition along with IC_50_ values of 16.21 μM/ml against SKOV-3 human cancer cell lines	Xu et al. (2018)
			Annexin-V/PI binding assay	Induction of 33.95% of apoptosis	
4	Epicatechin gallate	Nanoparticles	*In vitro* cytotoxicity assay SKOV-3 and OVCAR-3	IC_50_ values of 20 µM against SKOV-3 and 50 µM against OVCAR-3	Huh et al. (2004); Spinella et al. (2006); Chen et al. (2011); Singh et al. (2011); Yan et al. (2011); Huang et al. (2020); Qin et al. (2020)
			Western blotting	Downregulation of ET_A_R-dependent signaling pathways	
5	Fucosterol	Nanoparticles	Cell cycle	Cell cycle arrest in OV90 cells	Bae et al. (2020a)
			*In vivo* xenograft model	Reduces the tumor progression in zebrafish using the *in vivo* xenograft model	
6	Paclitaxel	Nanoparticles	MTT assay against ovarian cancer	IC_50_ values of 5.4 ng against SKOV-3-Luc	Xiao et al. (2009)
	Paclitaxel	Paclitaxel-loaded lipid nanoparticles	*In vitro* cytotoxicity assay	IC_50_ values of 2.5 mg/L against HEY cell lines	Zhai et al. (2018)
			*In vivo* xenograft model	50% reduction in tumor burden in BALB/c nude tumor-bearing mice at a dose of 5 mg/kg	
7	Resveratrol	Resveratrol-loaded liposomes	*In vitro* assay	It induces autophagy *via* activation of the expression of proteins sirtuin-1 and Akt/mTOR.	Sharifi-Rad et al. (2021)
			*In vivo* xenograft model	62% reduction in the tumor size in a dose of 200 mg/kg	
8	Sulforaphane	Sulforaphane–cisplatin nanoparticles	*In vitro* cytotoxicit*y* assay against human ovarian cancer cell lines	IC_50_ values of 40 μmol/L against SKOV-3 cell lines	Chaudhuri et al. (2007); Xu Y et al. (2019)
			Western blotting	It also induces apoptosis and inhibition of the cyclin D1, ckd6, and ckd4 levels	
9	Theaflavin-3,3ʹ-digallate	PLGA-loaded nanoparticles	Apoptosis assessment using cell cycle analysis	It induces arrest in the G2 phase	Jiang et al. (2021); Tu et al. (2016)
			Western blotting	Cleavage of caspase-3 and caspase-7	

## Future research and conclusion

Ovarian cancer is a major source of quality of life deterioration, mortality, and healthcare expenditure among females. The available treatment strategies are subject to a number of limitations that call for thorough investigation for novel therapeutic solutions. Phytochemicals provide researchers with a diverse pool of compounds capable of targeting different aspects of the disease’s pathogenesis. Correlating the chemical properties of natural compounds such as flavonoids and polyphenols with their reported preclinical and clinical effectiveness is essential in the effort to develop etiological treatments with favorable effectiveness and limited induced resistance and side effects. An innovative drug design can further improve these outcomes.

In the future and on the basis of sufficient clinical evidence, the applications of phytochemicals in ovarian cancer can be divided into two principal categories; their use as complementary regimens acting synergistically with the available chemotherapeutics or their use as standalone therapeutic regimens. The former pertains to reducing ER stress and the subsequent risk of disease relapse and metastasis. They can also be used to decrease the likelihood of drug resistance, provided that lower chemotherapeutic doses will be required during the simultaneous administration of natural compounds. Future research should investigate the potential of natural compounds to be integrated with hypothermic intraperitoneal chemotherapy (HIPEC). The application of drug formulations directly on the site of the tumor can help overcome several challenges related to drug metabolism. It can also decrease the relapse rate and the need to subject patients to numerous adjuvant therapy cycles (van Driel et al., 2018).

On the other hand, natural compounds might pave the way to novel chemotherapeutic agents, with a multipotent combination design, higher effectiveness, and lower drug resistance and adverse effect rates. ADME properties of natural products are found questionable in many cases, and that is where nanoformulation technologies can facilitate. Nanomaterials will not only be able to improve the ADME properties, especially bioavailability, but also contribute to the potentiation of the therapeutic activity in many cases (El-kharrag et al., 2012; El-kharrag et al., 2018; Xie et al., 2021). Scrutinizing relevant findings; sharing research questions, protocols, and methodologies; and promoting collaboration between the academia and industry can accelerate such developments. Certainly, the latter needs to be adequately regulated by research integrity bodies and healthcare authorities. Maintaining open networks of communication with policymakers, clinicians, and patients’ representatives can also serve as a compass for relevant research efforts. Communication with stakeholders ensures the flow of funding and regulatory support, interaction with clinicians helps refine research questions, and dialog with patients helps understand the practical drawbacks of conventional therapies that need to be addressed. Overall, such a research culture can strengthen confidence to the field and be transferred to other research and development sectors.
